# Parallel Selection on *TRPV6* in Human Populations

**DOI:** 10.1371/journal.pone.0001686

**Published:** 2008-02-27

**Authors:** David A. Hughes, Kun Tang, Rainer Strotmann, Torsten Schöneberg, Jean Prenen, Bernd Nilius, Mark Stoneking

**Affiliations:** 1 Max Plank Institute for Evolutionary Anthropology, Leipzig, Germany; 2 Institute of Biochemistry, Molecular Biochemistry, Medical Faculty, University of Leipzig, Germany; 3 Department of Physiology, Katholieke Universiteit Leuven, Leuven, Belgium; Indiana University, United States of America

## Abstract

We identified and examined a candidate gene for local directional selection in Europeans, *TRPV6*, and conclude that selection has acted on standing genetic variation at this locus, creating parallel soft sweep events in humans. A novel modification of the extended haplotype homozygosity (EHH) test was utilized, which compares EHH for a single allele across populations, to investigate the signature of selection at *TRPV6* and neighboring linked loci in published data sets for Europeans, Asians and African-Americans, as well as in newly-obtained sequence data for additional populations. We find that all non-African populations carry a signature of selection on the same haplotype at the *TRPV6* locus. The selective footprints, however, are significantly differentiated between non-African populations and estimated to be younger than an ancestral population of non-Africans. The possibility of a single selection event occurring in an ancestral population of non-Africans was tested by simulations and rejected. The putatively-selected *TRPV6* haplotype contains three candidate sites for functional differences, namely derived non-synonymous substitutions C157R, M378V and M681T. Potential functional differences between the ancestral and derived *TRPV6* proteins were investigated by cloning the ancestral and derived forms, transfecting cell lines, and carrying out electrophysiology experiments via patch clamp analysis. No statistically-significant differences in biophysical channel function were found, although one property of the protein, namely Ca^2+^ dependent inactivation, may show functionally relevant differences between the ancestral and derived forms. Although the reason for selection on this locus remains elusive, this is the first demonstration of a widespread parallel selection event acting on standing genetic variation in humans, and highlights the utility of between population EHH statistics.

## Introduction

Recently, with the advent of large, publicly-available data sets, the completion of many mammalian genome sequences, and large-scale genotyping resources, there has been much effort in attempting to identify loci which have potentially been under selection in the entire human species, or locally in specific human populations [1]–[11]. While there has been much success in producing lists of candidate genes, less effort has gone into a detailed characterization of the candidate genes, to verify the signature of selection obtained from genome-wide data, elucidate the functional differences between selected and non-selected alleles and their phenotypic consequences, and (ultimately) identify the nature of the selective force that produced the footprint of selection. Such further characterization is necessary for a complete understanding of the role and consequences of selection in shaping human genetic variation.

Here we present an analysis of independent parallel local selection on a candidate gene, *TRPV6*. *TRPV6* encodes a Ca^2+^ selective ion channel of the transient receptor potential (TRP) superfamily, which is critically involved in Ca^2+^ (re)absorption [12]. *TPRV6* is the primary route of dietary calcium uptake, and has previously been identified as a candidate for local selection in two other studies [1], [8]. These studies both used the SeattleSNPs data base, consisting at the time of full sequence data for over 130 genes in twenty-four individuals of African American decent and twenty-three individuals of European decent. Both studies identified putative selected genes by comparing the empirical data to simulated data that was based on an assumed demographic history. These studies found that the *TRPV6* locus has an excess of high frequency derived alleles, an excess of rare alleles, and reduced diversity in Europeans relative to African-Americans, all consistent with local directional selection in Europeans. Furthermore, the date of selection in Europeans was estimated to be around 10,000 years before present, suggesting that *TRPV6* may have coevolved with lactose tolerance [13] as fresh milk is a major source of calcium in European populations.

The *TRPV6* locus contains three non-synonymous substitutions (C157R, M378V and M681T) for which the derived allele is fixed (or nearly so) in Europeans, but are each polymorphic in African-Americans with a derived allele frequency of ∼50%. The lack of any unique high frequency allele in Europeans suggested that selection on *TRPV6* must have occurred on a “preexisting mutation in the ancestral African population…”, which subsequently “…became advantageous in a new environment and rose to high frequency” [8]. However, three genes flanking *TRPV6*, namely *EPHB6*, *TRPV5* and *KEL*, were also identified as potential target(s) of selection that could then be influencing genetic variation in neighboring genes through hitchhiking [1], [14]. Nonetheless, since *TRPV6* exhibits the most extreme departures from neutrality and is the only gene among these four with any high frequency derived amino acid substitutions, it is considered to be the most likely target of selection [1].

A subsequent study found that *TRPV6* exhibits accelerated protein evolution on the human lineage, as compared to Pan, Mus and Rattus [15]. A branch-sites model [16] identified six sites with a high posterior probability of having been subject to selection; three of these sites are the nonsynonymous substitutions for which the derived alleles define the putatively selected haplotype in Europeans [15]. This study also sequenced the three exons containing these non-synonymous substitutions in 90 individuals from six human populations, as well as genotyped these three sites in the CEPH diversity panel, a collection of DNAs from 51 human populations [17]. The three non-synonymous sites were fixed or nearly so for the derived alleles in all non-African populations, and each of the non-African populations exhibited reduced diversity as compared to sub-Saharan African populations, consistent with the action of selection [15]. This shared signature of selection among all non-African populations studied led to the suggestion that the selective event occurred around the time of the major human migration out of Africa some 60,000–100,000 years before present, and moreover indicated that dairying could not be the selective pressure [15].

In the present study we have also independently identified *TRPV6* as a candidate gene of local directional selection, using the SeattleSNPs data set and a different genome scan approach. We further characterize the footprint of selection on *TRPV6* in the aforementioned and additional populations, using a novel extended haplotype homozygosity approach, and find evidence for independent, parallel selection in Europeans and Asians (and possibly other populations). Our data and analyses suggest selection on standing genetic variation, also known as soft sweeps, in different populations for the same haplotype that differs from the ancestral haplotype by the three previously mentioned nonsynonymous substitutions. In addition, we also investigated the functionality of the ancestral form of *TRPV6* and the putatively-selected, derived form of *TRPV6*. Although some electrophysiological differences in protein function were found between the two forms in *TRPV6*, these differences were not statistically-significant. Thus, while functional consequences of the variation at *TRPV6* and the underlying selective force remain to be determined, this study does advance our knowledge about the temporal and spatial pattern of selection on *TRPV6*.

## Results and Discussion

### SeattleSNP Genome Scan, Neutrality tests, and Dating Time since Selection

We identified *TRPV6* as a putative target of local selection by implementing a genome scan based on two summary statistics of the sequence data (Figure S1), analogous to that performed previously [6]. A drastic reduction in diversity was identified at the *TRPV6* locus (Figure S2) and at the neighboring loci *EPHB6*, *TRPV5* and *KEL*. Additionally, statistically significant evidence for selection based on summary statistics of the allele frequency spectrum and the HKA test for each of these genes was found (Table S2). However, *TRPV6* exhibited the most significant departures from neutrality. Given these data and previous studies [1], [8], [15] we focused on the *TRPV6* locus as the putative site of selection. We then used the full sequence data from SeattleSNPs to estimate the time since fixation in Europeans, using two different methods. We estimated the age since fixation in Europeans to be approximately 7 ky (Figure S3). These results are comparable to a previous estimated date of 10 ky [1], but are not consistent with a suggested date of 60–100 ky [15], which was based solely on the apparent global distribution of selection at this locus. Finally, we also genotyped the CEPH diversity panel for the three non-synonymous substitutions (C157R, M378V and M681T) in a MALDI-TOF assay (Table S4) and confirmed near fixation for the derived allele at each of these sites in all non-African populations [15]. Further, discussion of these results, and the relevant materials and methods for these analyses, which confirm previous work, can be found in Supporting Information.

### Rab (Between Populations EHH) at *TRPV6* in Europeans

**Figure 1:**
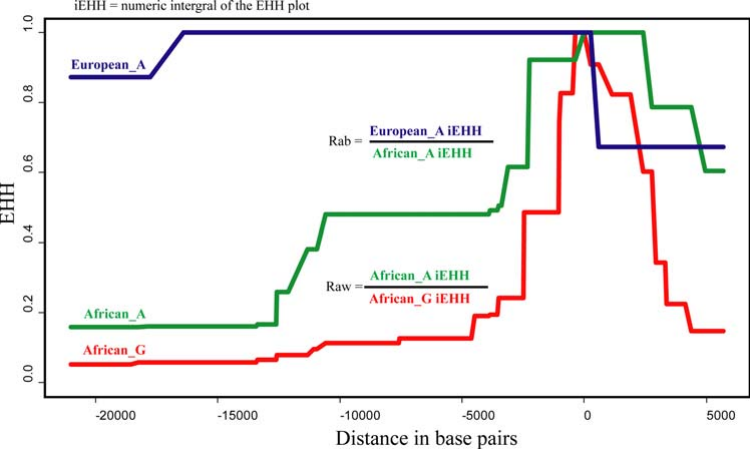
An EHH plot of the *TRPV6* locus from the SeattleSNP data, illustrating the calculation of Rab and Raw statistics. The core SNP is the M378V mutation, where allele A is derived and allele G is ancestral.

Recently, new methods have been developed for investigating loci for selection by evaluating the extended haplotype homozygosity (EHH) associated with an allele [10], [18], [19]. The premise of the EHH test is to evaluate the age of an allele [20] by comparing the decay of haplotype homozygosity among haplotypes with a pre-defined set of core SNPs. Haplotypes that have been subject to selection will have increased in frequency more rapidly than expected under neutrality, and hence will be associated with extended haplotypes relative to haplotypes that have not been subject to selection. A caveat and yet a useful feature of EHH tests is that discernable selective signatures may be found for selective events that occurred up to 400 generations ago [18] but are not expected for much more than 1,000 generations [10], [21]. Moreover, it has recently been shown that EHH tests have greater power to detect recent selection than other tests [10]. We utilized this methodology and calculated Raw values (relative iEHH of an allele within a population), which are based on comparing the EHH associated with the two different alleles at a SNP within a single population [19]. We also introduce a novel EHH statistic called Rab (relative iEHH of an allele between populations), which compares the iEHH (integrated EHH) of the same allele between two different populations (Figure 1, see Materials and Methods). The Rab statistic is directly analogous to the recently developed Rsb statistic (relative iEHH of a site between populations) [19], and allows us to analyze SNPs which are fixed (or nearly so) in a population and for which there is thus little information in a within-population comparison. Extreme Rab values would be associated with alleles that have potentially been under selection in one of the two populations. The median Rab value between Europeans and African-Americans is 1.54 in the SeattleSNP data, indicating that on average there is more EHH in Europeans than in African-Americans, consistent with previous findings of more linkage disequilibrium in Europeans than in African-Americans [22]–[24]. To determine if the *TRPV6* locus in Europeans has a preponderance of sites that have extreme Rab values with respect to African-Americans, we calculated the number of sites found above the 95^th^ quantile of the Rab distribution for each gene in the SeattleSNPs data set and in a simulated data set (see Materials and Methods). From the empirical distribution, 56.25% of the 112 *TRPV6* SNPs are above the 95^th^ percentile and 28.57% are above the 99^th^ percentile. The extent of the difference in EHH between Europeans and African-Americans can be seen in Figure 1. None of the other 220 genes in the SeattleSNPs dataset exceed *TRPV6* in terms of the number of SNPs with Rab values above either the 95^th^ or the 99^th^ percentile. Based on the quantiles of the simulated data of 10,000 loci, 58.92% (p-value = 0.0021) and 57.14% (p-value = 0.0001) of *TRPV6* SNPs are above the 95^th^ and 99^th^ quantile respectively. These analyses demonstrate that the EHH associated with *TRPV6* in Europeans is highly unusual when compared to both other genes in the SeattleSNPs data and to simulated data, suggesting that local directional selection has indeed influenced variation and haplotype homozygosity at the *TRPV6* locus in Europeans.

### Signature of selection on *TRPV6* in other non-African populations

**Figure 2:**
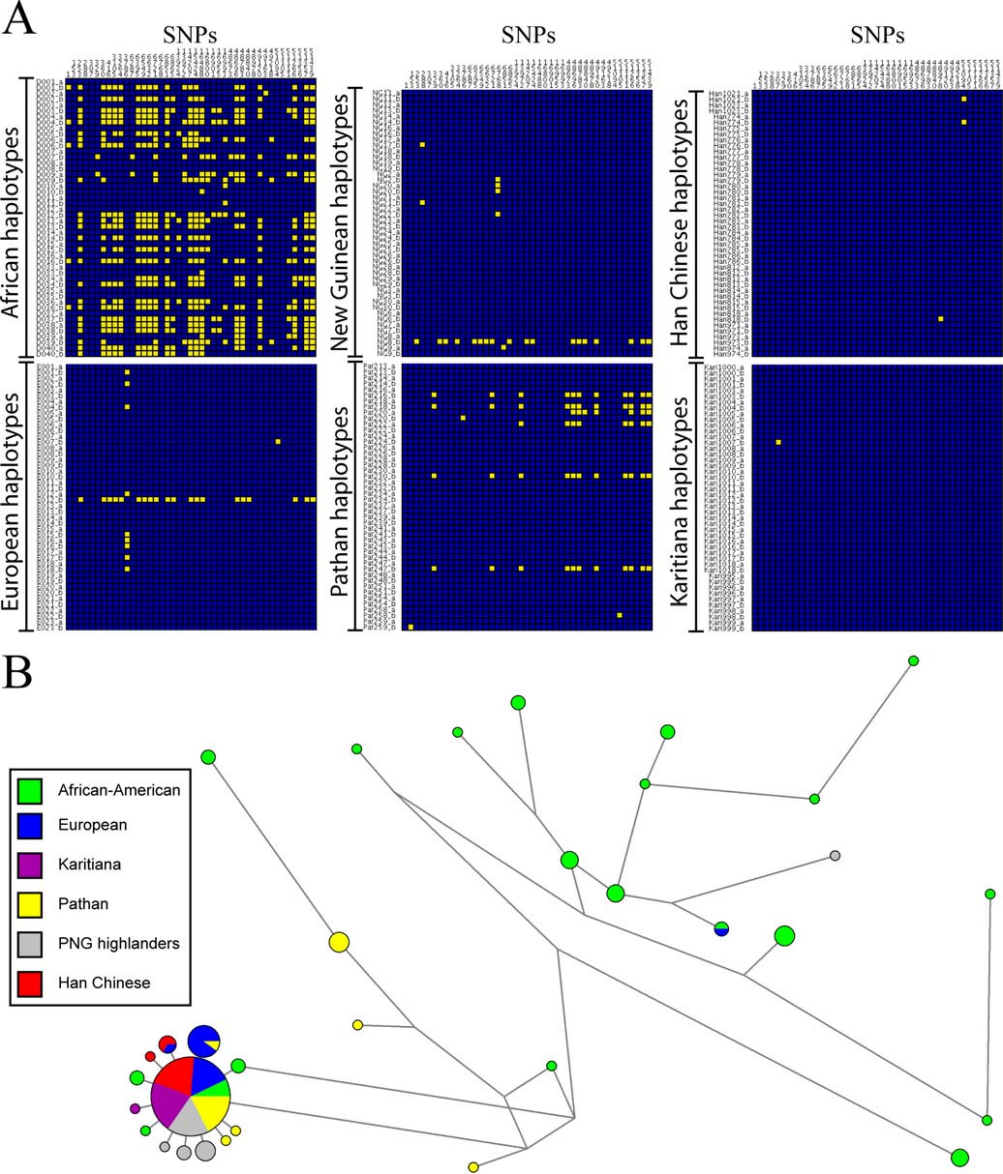
Visual haplotype graph and network for sequence data around the *TRPV6* locus in 6 populations. A) Visual haplotype graph of sequence data generated for four populations (New Guinea highlanders, the Pathan of Pakistan, Han Chinese, and the Karitiana of Brazil), compared to the European and African sequences from the Seattle SNPs dataset. B) A network of the sequence data from the Karitiana, Pathan, New Guinea highlanders, Han Chinese, Europeans, and Africans.

**Figure 3:**
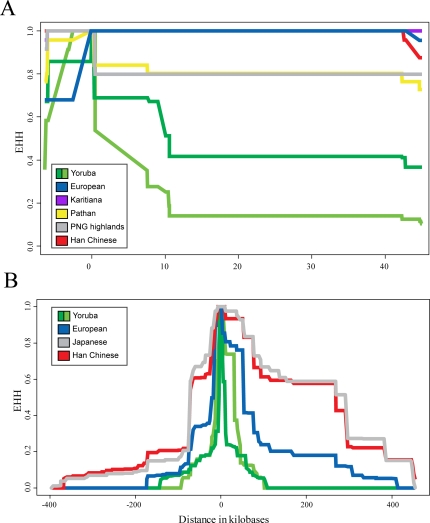
EHH decay plots for sequence data and HapMap data. A) An EHH decay plot with the C157R mutation as the core SNP from sequence data generated for the Karitiana, Han Chinese, New Guinea highlanders, and Pathan, along with data for Europeans and Africans (light green is the ancestral allele, dark green is the derived allele) from the SeattleSNPs dataset. Only the derived allele is plotted for each non-African population. B) EHH decay plot from HapMap data with the M378T mutation as the core. For the Yoruba, light green indicates the ancestral haplotype and dark green indicates the derived haplotype.

All non-African populations are fixed or nearly so for the derived, putatively selected haplotype in Europeans (see Figure S5); do they also show the same signature of selection as in Europeans? To address this question, we sequenced 4.1 kb of non-consecutive sequence in a 52 kb region around *TRPV6* (Figure S6) in four additional non-African populations: Karitiana from Brazil, Pathan from Pakistan, Han Chinese, and highland Papua New Guineans. As in Europeans, there is an extreme paucity of variation in these populations as compared to Africans (Figure 2), as well as large EHH when compared to the same alleles in Africans (Table 1). Moreover, each of these populations has haplotype homozygosity levels and Rab values that are comparable to or even greater than that found in Europeans (Figure 3A; Table 1). Strikingly, the polymorphisms in this 4.1 kb of sequence include only one singleton and one doubleton in the Han Chinese, only one singleton in the Karitiana, the presence of a single African-like chromosome in highland New Guinea (as also found in Europeans), and the presence in the Pathan of two distinct haplotypes both carrying the derived, putatively-selected alleles, possibly as a result of a recombination event prior to or early in the selective phase (Figure 3A). These results indicate that local directional selection has acted on each of these populations, and by extension in all non-African populations, as it has in Europeans.

**Table 1:** Summary statistics for approximately 4.1 kb of sequence data in and around *TRPV6* for six populations.

Population	S	θ	h	Hd	π	k	Rab
African	34	7.66	20	0.889	0.285	12.269	NA
European	22	5.01	4	0.39	0.029	1.23	2.44
New Guinea	21	4.78	5	0.314	0.025	1.07	2.49
Pathan	14	3.19	5	0.278	0.05	2.13	2.35
Han Chinese	2	0.455	3	0.127	0.003	0.129	2.78
Karitana	1	0.228	2	0.043	0.001	0.043	2.82

European and African data are from the SeattleSNPs data, corresponding to the regions sequenced in highland New Guineans, Pathans, Han Chinese, and the Karitiana. S is the number of segregating sites, θ_W_ is Watterson's theta or the population mutation rate based on S, h is the number of haplotypes, Hd is haplotype diversity, π is nucleotide diversity, k is the average number of nucleotide differences, and Rab is the average Rab value when the iEHH for that population is compared against the iEHH for Africans.

### EHH and Rab at *TRPV6* in HapMap data:

To further investigate the putative selection on *TRPV6* in non-Africans, we analyzed the EHH patterns in the HapMap data [25], which provides an analysis of EHH over a much larger physical distance for Europeans and Africans (Yoruba) than with the SeattleSNPs data, as well as the addition of two Asian populations (Han Chinese and Japanese). We calculated Rab values using HapMap Phase I data for each non-African population compared to the Yoruba for approximately 20,000 genes. We then determined the proportion of sites which were above the 95^th^ percentile for each gene and compared these to *TRPV6* and neighboring genes (Table 2). In the two Asian groups, the *TRPV6* locus had a significant proportion of SNPs that were above the 95^th^ percentile of their respective Rab bins, while Europeans were nearly significant. None of the other genes closely-linked to *TRPV6* were significant in this test, although they do harbor alleles which have significant Rab values. Previously, the region surrounding *TRPV6* (including *TRPV5* and *EPHB6*) was identified as a target of selection using the analogous Rsb statistic [19]. This difference most likely is because our analysis focuses on individual genes, whereas the previous study identified larger genomic regions that had been influenced by local selection [19].

**Table 2:** Rab significance values for *EPHB6*, *TRPV6*, *TRPV5*, and *KEL* in pairwise population comparisons from the HapMap data.

Locus	Europe/African	Han Chinese/African	Japanese/African	Han Chinese/European	Japanese/European
*EPHB6*	0%, 1	16.67%, 0.093	9.09%, 0.154	27.27%, 0.048	40%, 0.030
*TRPV6*	25%, 0.056	75%, 0.006	50%, 0.022	37.5%, 0.032	33.3%, 0.041
*TRPV5*	0%, 1	15.78%, 0.095	15.79%, 0.096	31.58%, 0.040	47.4%, 0.02123
*KEL*	18.18%, 0.078	9.09% , 0.154	9.09%, 0.154	25%, 0.061	25%, 0.0633

The first value is the proportion of sites in each gene which are found above the 95% quantile, followed by p-values for that gene as compared to all other genes.

Remarkably, the EHH around *TRPV6* extends more than 820 kb in Asians (both Chinese and Japanese), compared to 582 kb in Europeans and 190 kb in the Yoruba (Figure 3B). This raises the question as to whether the signal of selection at *TRPV6* differs between Europeans and Asians. We therefore compared the iEHH of each allele between the Asians and Europeans; the Rab values are significant (or nearly so) for all four genes in the TPRV6 region when either Asian group is compared to Europeans, as compared to other genes in the HapMap phase 1 data (Table 2). On average, the amount of EHH found in Han Chinese and Japanese at the *TRPV6* locus is 2.18 and 2.39 times greater than that in Europeans, and both are 8.23 times greater than that in Africans. Thus, the *TRPV6* region in Asians has a significant excess of alleles with significant Rab (iEHH), as compared to the remainder of the genome and to either Africans or Europeans.

### Parallel Selection events or a Single Selective Event

Although it was previously hypothesized that selection on *TRPV6* occurred in a population ancestral to all non-African populations[15], the significant Rab values obtained when comparing Asian populations to European, as well as the estimated time since fixation in Europeans of 7 ky, raises the intriguing possibility that selection may have occurred independently in Asians and Europeans, with stronger and/or more recent selection in Asians. We therefore used simulations to investigate if the observed differences in EHH between Europeans and Asians (Rab of ∼2.3) could be explained by a single selection event which occurred in the ancestral population of Europeans and Asians, some 50 ky [26]–[32], as suggested previously [15]. Using Hudson's ms program [33] and demographic parameters previously found to provide the best fit to human data [30] we simulated 850kbp segments (roughly the length needed for EHH in Asians to reach 0.05) for Africans, Europeans and Asians. To simulate a very strong selective event, we introduced a severe bottleneck in the ancestral population of non-Africans, setting the effective populations size (Ne) to 1 at 50,100 years before present for five generations. This would have the same effect as a selective event, in that a single haplotype will be fixed in this ancestral population, prior to the split of Europeans and Asians. This scenario provides a maximum amount of opportunity for the subsequent independent demographic histories of Asian and European populations to accumulate mutations and recombination events, which may then recapitulate the observed difference in EHH (Rab) seen in the empirical data. Our null hypothesis is that these independent demographic histories could, following a shared selective event, explain the observed difference in EHH between Asians and Europeans; rejecting this null hypothesis would imply independent selection for the same haplotype in Asians and Europeans. Average Rab values across all sites and all simulations were 1.08 for Asians as compared to Europeans, stressing the similarity in EHH between the two populations. As expected following a selective sweep, large Rab values were observed between non-Africans (who experienced the simulated selective event) and Africans (who did not experience the selection): average Rab equals 6.42 for Asians as compared to Africans, and 6.41 for Europeans compared to Africans. Moreover, a significant difference was observed between empirical and simulated Rab values between Europeans and Asians (p = 0.023, see Materials and Methods). These results suggest that the observed, significantly different EHH between Asians and Europeans is unlikely to reflect a single selection event in an ancestral population of these two populations some 50,000 years ago. Rather, we conclude from the data that the same *TRPV6* haplotype was subject to more recent, and hence independent, parallel selection in these different populations, with stronger and/or more recent selection in Asians than in Europeans.

### Selection on *TRPV6* in Africans

Allele frequency and the HKA tests revealed no significant deviations from neutrality in African-Americans (Table S1), however there were a large number of African-American haplotypes which were closely related to the star-shaped cluster of European haplotypes (Figure 2B). Although this may reflect European admixture, we also observed the selected haplotype at moderate to high frequency in some populations of Africa (Figure S5). Thus, is there any signal of selection on *TRPV6* in African populations?

We first investigated EHH at the *TRPV6* locus in African-Americans by calculating Raw values [19] for the SeattleSNPs African-American data; these Raw values were then compared to both the empirical distribution of Raw values for all genes in the Seattle SNPs data for African-Americans, and to a simulated distribution. The proportion of Raw values above the 95^th^ percentile approaches statistical significance when compared to the empirical SeattleSNPs distribution (p = 0.064 with 12.9% of 109 sites significant), and is highly significant when compared to the simulated data, with 41.3% of the 109 sites above the 95^th^ quantile (p = 0.012) and with 22% of the sites above the 99^th^ quantile (p = 0.006). Since this signature of selection could reflect European admixture in the SeattleSNPs African-American dataset, we also calculated Raw statistics for each SNP for the Yoruba from the HapMap data. No allele at the *TRPV6* locus in the Yoruba has a significant Raw value. The signature of selection that is present in the SeattleSNPs data therefore is likely to be the result of admixture with individuals of European origin, or (less likely) reflects very recent selection in this African-American population.

It was previously reported that the Mozabite population of North Africa has an excess of high frequency derived alleles and a significant Fay and Wu's H value [15], which is consistent with the high frequency of the putative-selected haplotype in this population (Figure S5). However, this could be explained by admixture of this North African population with Europeans, and not necessarily selection operating on this population. Furthermore it was also previously suggested that South Africans and African Americans may be experiencing some form of balancing selection at the *TRPV6* locus, based on large positive Tajima's D values [15]. Alternatively, given the data presented here, these large positive Tajima's D values could reflect an incomplete sweep that is in the process of driving some alleles to moderate frequency, while others are decreasing in frequency. Another possibility is the action of a soft sweep (i.e. selection on standing variation), shortening EHH, creating spurious values in neutrality tests, and in general confounding the signature of selection [34], [35]. It would be necessary to obtain more sequence data in and around the *TRPV6* locus from more African populations, to determine if *TRPV6* has experienced non-neutral evolution in African populations, while considering the possible confounding affects of a soft sweep.

### Functional analysis of *TRPV6* haplotypes

The putatively-selected *TRPV6* haplotype is defined by the derived state of three amino acid substitutions: C157R, M378V, and M681T. The presence of three amino acid substitutions on a recently selected haplotype suggests that one (or more) of these substitutions may result in some phenotypic difference that may then be influenced by selection. We therefore investigated possible functional differences between the ancestral and derived *TRPV6* proteins by cloning each form into expression vectors and transfecting the constructs into a human cell line; we then used patch clamp analysis to investigate the electrophysiology of the *TRPV6* calcium channel in cells expressing either the ancestral or derived *TRPV6* proteins. Our functional screening comprised the most typical features of *TRPV6*, namely, the strong inward rectification (i.e. the inward currents are much larger than outward currents) [36], the very positive reversal potentials if Ba^2+^ or Ca^2+^ are the charge carriers (indicating high selectivity for both cations), block by Mg^2+^ of *TRPV6* activity, and Ca^2+^ dependent inactivation.

**Figure 4:**
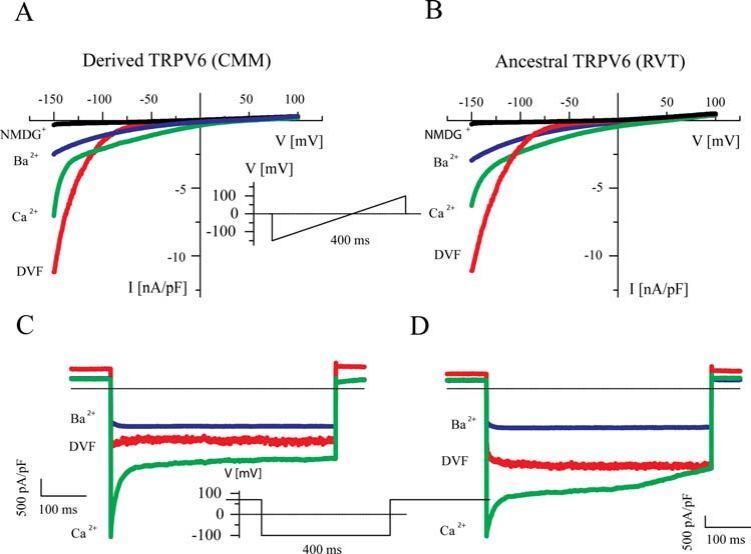
Current–voltage relationships (IV curves) for the derived (CMM) and ancestral (RVT) forms of *TRPV6.* A) IV curves for the derived form obtained from the voltage ramps shown in the inset. Note the strong inward rectification and the shift of the reversal potentials when Ca^2+^ or Ba^2+^ are the charge carriers (DVF: divalent free solution, Ca^2+^: 30 mM CaCl_2_ with NMDG^+^ as substitute for Na^+^, see methods for details). B) IV curves for the ancestral form, using the same protocol as in A. C) Current activation for the derived *TRPV6* form by voltage steps from +70 mV to −100 mV as shown in the inset. Note the fast initial inactivation when Ca^2+^ is the charge carrier and the absence of current decay in DVF conditions and with Ba^2+^ as the charge carrier. D) Current activation for the ancestral *TRPV6* form, with the same protocol as in C.

The intracellular expression pattern of both constructs was assessed by epifluorescence microscopy and revealed largely plasma membrane-bound CFP fluorescence, compatible with surface expression of the proteins. No differences in the expression levels or surface expression of derived and ancestral *TRPV6* were observed (data not shown). Figure 4 shows a typical patch clamp experiment in which a voltage ramp protocol was used under different ion conditions. In the absence of any divalent cations in the bath solution, e.g. with the current carried by Na^+^, large and strongly inward rectifying currents were measured which reversed close to 0 mV. This is the typical hallmark of *TRPV5* and *TRPV6* currents [37]–[39]. However, when Ca^2+^ or Ba^2+^ were the only charge carrier for inward currents, the current density increased and the reversal potentials were shifted to more than +50 mV, indicating high selectivity for these divalent cations [37]. This experiment shows that major changes in permeability cannot be expected between derived and ancestral *TRPV6*. This is not unexpected because the pore region, which is responsible for this unique permeation pattern [36], [40], [41], is the same in the two versions of *TRPV6*. A fast initial decay was observed when Ca^2+^ was the charge carrier (Figure 4A). This fast decrease in the Ca^2+^ entry is due to channels closing, i.e. inactivation, a process which is mediated by Ca^2+^ itself entering the cell through the channel and is known as Ca^2+^ dependent inactivation [12], [39], [42], [43]. Ca^2+^-dependent inactivation acts in the sense of a negative feedback on Ca^2+^ entry through the channel. This functional fingerprint was also observed for the ancestral construct (Figure 4B). By using voltage steps from +70 mV to −100 mV, inactivation of currents through *TRPV6* was measured. If Ca^2+^ is the charge carrier, an initial fast inactivation is followed by a slower decay (Figure 4C). In divalent-free solution (DVF) and with Ba^2+ ^as the charge carrier, no inactivation was observed during the first 400 ms. This behavior is again typical for the Ca^2+^-dependent inactivation of *TRPV6*, as described in detail previously [43]. An identical behavior was observed for the ancestral construct (Figure 4D).

**Figure 5:**
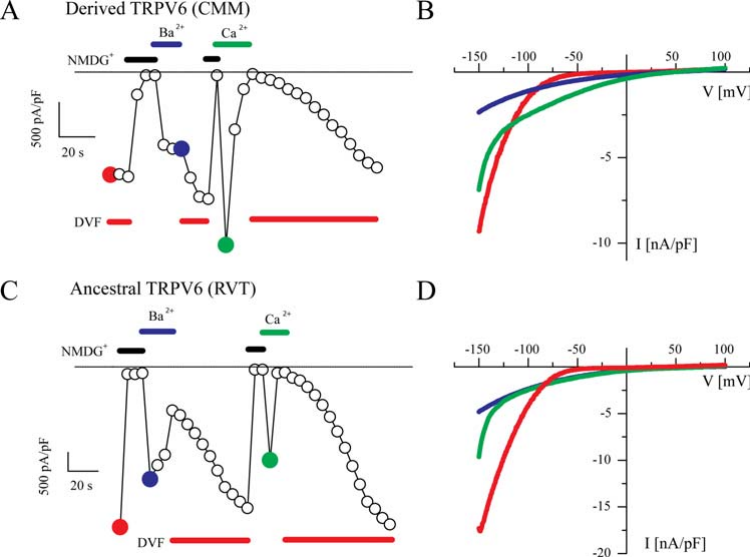
Time course of *TRPV6* currents with changing extracellular ionic conditions. A) Current density for the derived *TRPV6* form at −100 mV obtained from voltage ramps. Bars denoted DVF indicates superfusion of the cell with a divalent free solution; bars above the time course indicate changes to NMGD^+^, Ba^2+^ and Ca^2+^. Note the typical large currents in DVF, the complete inhibition of inward current with NMDG^+^, the relatively stable current in Ba^2+^, and the fast decay with Ca^2+ ^as the charge carrier. Also note the slow recovery from Ca^2+^ dependent inactivation. B) IV curves from the time course shown in A, measured at the times which are indicated by solid points (red DVF, blue Ba^2+^, green Ca^2+^). C and D) Identical experiments as in A and B, respectively, with the ancestral *TRPV6* form.

The typical response to changes in the extracellular solution for the ancestral and the derived construct are shown in Figure 5. Large currents were observed in ramp protocols at −100mV in DVF solution. Substitution of all extracellular cations by the non-permeable N-methyl d-glucamine (NMDG^+^) abolished the inward current. If the solution was changed to Ba^2+^ as the only charge carrier, the current increased again and remained stable. Application of a solution with Ca^2+^ as the only charge carrier induced a transiently increased current, which rapidly decayed (Figure 5A). The respective IV curves obtained from voltage ramps are shown in figure 5B and represent the typical IV fingerprint shown in figure 4A. The same pattern was observed for the ancestral construct: large inward currents in DVF solution, relative stable or only slowly decaying currents with Ba^2+^ as charge carrier, and rapidly inactivating current when the charge carrier changed to Ca^2+^ (Figure 5C). Also, the respective currents obtained from the time course are similar to the ones shown in figure 4B.

**Figure 6:**
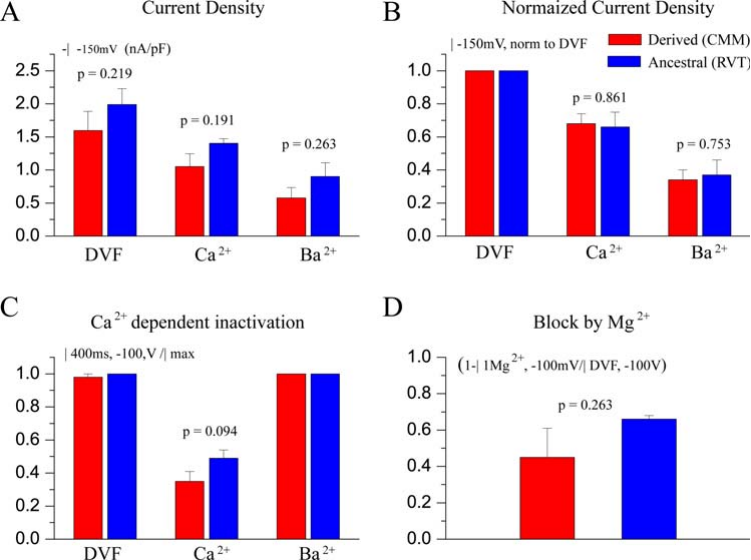
Analyses of three key feature of *TRPV6*: current density, Ca^2+^ dependent inactivation and block by Mg^2+^. Data from between 3 and 5 cells for all graphs (except where stated otherwise) with p values are indicated. A) Current density was measured at −150 mV under three ionic conditions for derived *TRPV6* and for ancestral *TRPV6*. DVF: divalent free (13 derived and 12 ancestral cells measured); Ba^2+^, Ca^2+^ refer to these divalent cations as the only charge carrier for the inward currents. Note that high current densities have been measured indicating high functional expression of the channels. B) Data were taken only from the same cells in which all three condition (DVF, Ba^2+^, Ca^2+^) could be measured (see protocol panel A). Current densities were normalized to the current in DVF solution at −150 mV. C) Ca^2+^ dependent inactivation was estimated by the ratio of the currents at the end of the 400 ms voltage step and the maximal inward current during the step (I_400ms,−100mV_/I_max_). Eleven derived and 13 ancestral alleles were tested for the effect in which Ca^2+^ has on Ca^2+^ dependent inactivation D) Block of *TRPV6* by Mg^2+^ was estimated from step protocols. I_1Mg2+,−100mV_/I_DVF,−100mV_ is the ratio of the maximal inward current measured from the same cell after changing from DVF to addition of 1 mM Mg^2+^. (1-ratio) gives the inhibition.

To compare these functional data statistically, we measured several parameters. First, current density at −150mV did not differ significantly between the derived and ancestral *TRPV6* proteins under all three conditions: DVF; charge carrier Ca^2+^; or charge carrier Ba^2+^ (Figure 6A). Second, the same holds when only data were taken from cells in which all three protocols could be measured (DVF, Ba^2+^, Ca^2+^) and the current density with Ca^2+^ and Ba^2+^ as the charge carrier could be normalized to the monovalent (DVF) current at −150 mV (Figure 6B). Third, as an estimate for Ca^2+^ dependent inactivation, we compared the ratio of the currents at the end of the 400 ms voltage step to −100 mV and the maximal inward current during the step (I_400ms,−100mV_/I_max_) for derived and ancestral *TRPV6*. This ratio reflects the extent of negative feedback inhibition of *TRPV6* by Ca^2+^. No inactivation is present with Ba^2+^ and under the DVF condition. In the presence of 30 mM Ca^2+^, inactivation of 48.9 +/− 5% and 34.9 +/− 6% was observed for the ancestral and derived *TRPV6* proteins. This difference in inactivation has a low, albeit not statistically-significant, probability of occurring by chance (p = 0.094, Figure 6C); however, this difference could be biologically significant as feedback inhibition and expression are together perhaps the most important regulators of the protein. Continuing, it has been shown in detail that extracellular Mg^2+^ blocks both *TRPV5* and *TRPV6* currents [12], [38], [44], [45]. We have therefore, fourth, measured the current decrease when 1 mM Mg^2+^ was added to the DVF solution in voltage step experiments. Currents were inhibited by ∼50% and again no significant difference between the ancestral and derived forms was observed (Figure 6D).

One of the amino acid differences between the derived and ancestral versions of *TRPV6*, C157R, is located between the third and the fourth ankyrin repeats of *TRPV6* (see Figure S4), which have been shown to be functionally important for multimerization, may initiate a molecular zippering process for protein-protein interaction, and may create an intracellular anchor necessary for functional subunit assembly [46]. Therefore, a C157R mutation could be functionally-significant in protein-protein interactions, and we cannot exclude at this point that protein-protein interactions might differ between the derived and ancestral versions of *TRPV6*. However, channel function per se is not significantly changed by the C157R mutation. It is more difficult to predict any functional changes for the M378V mutant, which is located extracellularly, in the loop between the first and the second transmembrane helix, TM1 and TM2. The residue M681T is located in a functionally important region, very close to the putative Ca^2+^/calmodulin binding site between residues 691 and 711 [43]. Ca^2+^/calmodulin binding to this region initiates Ca^2+^-dependent fast inactivation of *TRPV6*, which can be reversed by PKC dependent phosphorylation [47]. It is this feature of the two protein alleles which may be functionally relevant. The data therefore suggest the possibility that the ancestral form may be a better Ca^2+^ re-absorber than the putatively selected form of *TRPV6*, i.e the derived allele is more sensitive to Ca^2+^ feedback. Nonetheless, the differences we find in biochemical/biophysical function of the *TRPV6* protein (expressed as homotetramers) between the two forms are not statistically-significant. It is also possible that other aspects of *TRPV6* could still be affected, such as the putative site of protein-protein interaction in the N-terminus or the site close to a region which is critical for Ca^2+^ dependent inactivation [43]. Or, it may be that the amount of *TRPV6* channel differs between these two forms, because of expression or stability differences *in vivo*. Despite these extensive electrophysiological experiments, the phenotypic consequences of these different *TRPV6* haplotypes remain enigmatic.

### Conclusion

*TRPV6* is an epithelial calcium channel which is thought to be the primary protein in the absorption of calcium in the intestine and hence is key to absorbing calcium from the diet [44], [48]. This, and the proposed date of selection of about 10,000 years before present, led to the hypothesis that this locus may have co-evolved with lactase persistence with the onset of dairying in Europeans [1]. However, both this work and previous work [15] find signatures of selection in all non-African populations examined; there is thus no correlation between dairying and selection at this locus.

We conclude that the best explanation for the observed patterns of variation at *TRPV6* in non-African populations is the result of independent, parallel local selection acting on standing genetic variation (a soft sweep), analogous to the parallel evolution recently described in sticklebacks [49]. The putative haplotype is present in all tested populations of the world, consistent with this haplotype being present in the ancestral population of all humans. The selected allele therefore must have been at a high enough frequency to survive population bottlenecks during human migrations, but also at a low enough frequency so that it did not become associated with multiple haplotypes during the selection phase, thereby reducing or even eliminating the signature of selection [35], [50]–[52].

Bottlenecks such as those that non-Africans are thought to have experienced will remove haplotypes at low frequency, so that subsequent soft sweeps may start from alleles carried on a single haplotype [35]. While there is good evidence that the migrations of modern humans out of Africa were accompanied by bottlenecks [28], [30], [53], the extent to which such bottlenecks would have been sufficient to remove existing multiple haplotypes depends upon the frequency at which the standing genetic variation is present prior to the bottleneck and the magnitude and length of the bottleneck. In the SeattleSNPs data there is evidence suggesting the presence of multiple haplotypes in Europeans at the start of or during the sweep. At the *TRPV5* locus (which is 22 kb from the *TRPV6* locus) there are two major haplotypes present in Europeans that differ at 9 sites, all of which are also polymorphic in Africans, and thus by inference were present in the ancestral population (Figure S7). These two distinct haplotypes could be explained either by the presence of multiple haplotypes on the background of the selected allele prior to the onset of selection, or a recombination event which occurred early during the selective phase.

The timing of the selection event in Europeans and the apparent parallel, independent local selection in Europeans and Asians suggest that independent selective events may have also occurred in the Americas and in highland New Guinea, since both of these regions were colonized well before 7,000 years ago [54]–[56]. Moreover, the greater extent of EHH observed in Han Chinese and Japanese suggests that the selective event must have been more recent in time and/or stronger than that of Europeans.

Our analysis of several electrophysiological characteristics of *TRPV6* demonstrated no significant differences between the two alleles. However, the observed difference in Ca^2+^-dependent inactivation may nonetheless be biologically relevant. If it were, then the derived form of *TRPV6* would be quicker to “close” in response to Ca^2+^ feedback, signifying that it would reabsorb less Ca^2+^ than the ancestral form. Nonetheless, it is possible that the ancestral and derived forms differ not in electrophysiological characteristics, but rather via novel protein-protein interactions in the intracellular domains, or differences in the regulation of gene expression. These properties should be explored in the future. We further note that *TRPV6* not only absorbs calcium from the diet, but also increases cell proliferation of a variety of different cancer cells [57]–[59], as well serving an essential role in the differentiation of kerationcytes via a Ca^2+^ gradient in skin tissue [60]. This is an intriguing relationship due to the transcriptional regulation of *TRPV6* by 1,25-dihydroxyvitamin D3 [61], which is in turn derived from cholesterol via ultraviolet light. Since the availability of light is regulated by the amount of melanin in the skin, and the distribution of melanosomes throughout the skin is regulated by kerationcytes [62], [63], there is potentially a complex regulatory circuit involving *TRPV6*.

A potential selective force that could result in parallel selection in different populations is agriculture. The independent development of agriculture in several different parts of the world within the past 11,000 years [64] including the Middle East (11,000 ybp), East Asia (9,000 ybp), the Americas (5,000–4000 ybp), and the New Guinea highlands (9,000–6,000 ybp), fits remarkably well with the demonstrated parallel selection on *TRPV6* in the ancestors of Europeans and Asians, and potentially in the Americas and New Guinea as well. It may be that dietary changes related to the switch to agriculture underlie the selective event, or that selection on *TRPV6* is related to resistance to a particular disease that became important with the increased population densities associated with agriculture.

As a simple test of this hypothesis, we included three hunter-gatherer groups from India in our genotyping of the three tagging SNPs in worldwide populations (Koragas, Mullukurunan, and Mullukurumba; Table S3); however, all three populations were fixed for the derived, putatively-selected haplotype, suggesting that selection for this haplotype also occurred in the ancestors of these groups. While to the best of our knowledge these populations have always been hunter-gatherers, it is possible that they have reverted from an agricultural to a hunting-gathering lifestyle, as has been documented for other hunting-gathering groups [65]. Genotyping of additional hunter-gatherer groups would therefore be desirable.

In sum, in the absence of any current demonstrable functional difference between the ancestral and putatively-selected, derived form of *TRPV6*, the reason(s) for selection on *TRPV6* remain(s) enigmatic. However we have demonstrated that the most parsimonious explanation for the observed variation in humans at *TRPV6* is independent parallel soft sweeps occurring multiple times in human history. This is the first record, to our knowledge, of parallel selection acting on standing genetic variation in humans and suggests, as others have observed [10], that this may not be uncommon in recent human adaptive history.

## Materials and Methods

### Public Data Resources

We downloaded data from the SeattleSNPs Variation Discovery Resource at http://pga.mbt.washington.edu/on April 26, 2005 for 221 genes, of which 200 were sequenced from panel 1 and 21 sequenced from panel 2. The samples from panel 1 include 24 African-Americans from the Human Variation Panel and 23 European-Americans, while the samples from Panel 2 are HapMap samples [25], consisting of 24 Yoruba from Ibadan, Nigeria, and 23 CEPH European-Americans. Introns, exons, and UTRs were sequenced for each gene in each sample and in one *Pan troglodytes*. HapMap Phase I data as well as individual genotype data was downloaded in bulk from the International HapMap Project. HapMap data consists of genotype and phased data from four populations: 30 trios from the Yoruba people of Ibadan, Nigeria; 45 unrelated Japanese individuals from the greater Tokyo area; 45 unrelated Han Chinese from Beijing; and 30 trios from Americans of Northern and Western European origin.

### Extended Haplotype Homozygosity

Extended haplotype homozygosity (EHH) values [18] were calculated for every allele in each gene in the SeattleSNPs dataset using the inferred haplotypes from Phase v 2.0, obtained from the SeattleSNPs website. Following previous procedures [10], [19] we defined every SNP allele as a core and calculated the decay of EHH as the probability of selecting two identical haplotypes, starting with a value of 1 at the core site and decaying to 0 as the distance from the core site increases. To summarize the decay of EHH we calculated the integrated area under the EHH curve (iEHH) for each SNP allele. iEHH values were then used to calculate two relative EHH statistics, Raw (relative EHH of an allele within a population) and Rab (relative EHH of an allele between populations). Raw is a within-population statistic and is defined as the ratio of the iEHH values for the haplotypes defined by the two alleles at each SNP, as proposed previously [18]. Raw was calculated for each SNP, with the allele with the larger iEHH in the numerator. We introduce here a new between-population statistic, Rab, which is defined as the ratio of the iEHH values for the same core SNP allele in two different populations. Rab was calculated for every allele in the data with the European or non-African iEHH in the numerator. To determine if a locus has a preponderance of large Rab or Raw values, we calculated an empirical distribution of Rab and Raw values based on the other 220 genes in the SeattleSNP data, as well as generating Rab and Raw values for a simulated data set. For the empirical analysis we used the Phase v2.0 [66], [67] files that were supplied on the SeattleSNPs website, in which all sites with a minor allele frequency below 5% were removed prior to the Phase analysis. Rab and Raw values were calculated for every allele and binned according to the frequency of the European allele, or in the case of Raw according to the allele with the largest iEHH value. Alleles that did not occur at least twice in both populations were removed from the analysis and the 95% and 99% percentiles for each frequency bin were determined. For each gene we then determined the proportion of sites that were above each of these percentiles.

### Neutral Simulations

For the comparison to simulated data, we used Hudson's ms program [33] to simulate 10,000 loci with demographic parameters based on a model previously found to provide the best fit to human data [30], [33]. This model includes three populations (Africans, Europeans, and Asians), allows for migration between all populations, and includes a bottleneck for both non-African populations and a post-agricultural population expansion for each population. In the simulations used in EHH analyses (Rab and Raw) we chose a theta value for the simulations that yielded a median value of the observed number of segregating sites in *TRPV6* (146) and a recombination rate of 0.00103/base across the entire sequenced region. The recombination rate used here is the median recombination rate for African Americans, estimated by a previous study of the SeattleSNPs data [68]. In simulations testing neutrality based on the allele frequency spectrum, we used the observed number of segregating sites observed in the population under question.

### Selection Simulations

Simulations of a selective sweep were carried out as described above, with the following exceptions: migration between populations was excluded; the sequence length was set to 850kb; and a bottleneck was simulated by setting Ne to 1 for 5 generations starting 50,100 ybp for the ancestral population of Asians and Europeans. Simulations were based on the theta value observed in the African-American sequence data. This creates a drastic bottleneck equivalent to the effect of an instantaneous selective sweep. After five generations Ne was increased to 7700 and 50 generations later the European and Asian populations split and followed subsequent independent demographic histories with no migration. To simulate the ascertainment bias in the empirical data, each simulation included an additional 28 chromosomes, comprised of 4 African, 5 European and 5 Asians, from which a discovery panel was created to identify SNPs which were then used in the subsequent analysis. To determine if the eight empirical *TRPV6* Rab values are greater than those simulated, we performed two conservative one-sided Mann-Whitney U tests. The first compares all sites within the window in which *TRPV6* would lie for each simulation to the distribution of observed HapMap Rab values, while the second compares the top eight sites in each simulation from the previous test that also have an allele frequency greater than 0.95. We used this more stringent frequency cutoff because SNPs in the empirical data were also fixed or nearly so. P values were calculated as the proportion of simulations that had a Mann-Whitney U p-value greater than 0.05.

### DNA Amplification for Sequencing and Genotyping

25 µl PCR reactions were carried out in MJ Research Thermal Cyclers (MR Research, Waltham, MA, USA). Each PCR consisted of an initial DNA denaturation and *Taq* activation step at 95° for 15 min followed by 34 repeated cycles of denaturation at 95° for 1 min, an annealing step for 1 min and extension at 72° for 1 min. After amplification there was a final extension step at 72° for 10 min. The reactions included 20 ng of template DNA, 1× PCR buffer (Applied Biosystems, Foster City, CA, USA), 200 µM dNTPs (Amersham Biosciences, Uppsala, Sweden), 400 nM of each primer (Biotez, Berlin, Germany) and 1.25 units of AmpliTaq Gold DNA polymerase (Applied Biosystems, Foster City, CA, USA). All primers were designed with Primer3, and in cases of multiplexing the primers were further analyzed with NetPrimer.

### Additional Sequencing

Approximately 4.1 kb of non-consecutive sequence in a 52 kb region around *TRPV6* (Figure S6) was obtained from four additional populations: 23 Karitiana from Brazil, 23 Pathan from Pakistan, and 23 Han Chinese, all from the HGCP-CEPH Diversity Panel [17]; and 23 highland Papua New Guineans [69]. Regions for sequencing were selected to capture informative polymorphic sites, based on the data from SeattleSNPs. Primers are provided in Table S5. Sequences were aligned, concatenated and phased with Phase v2.0 [66], [70] and then iEHH and Rab values, with Africans as the comparative population, were calculated for each site in each population. Each PCR product was subsequently sequenced in both directions in a total volume of 10 µl. PCR amplification primers (Supplemental Table 5) were used as sequencing primers in reactions consisting of 1 µl of Applied Biosystems Big Dye Terminator v 1.1, 0.75× sequencing buffer, 320 nM primer (Biotez, Berlin, Germany) and 2ng of amplified template DNA. Sequencing was carried out in an ABI 3730 DNA Analyzer. Sequence data was analyzed manually with Applied Biosystems SeqScape Software V2.5 and reference sequences (Applied Biosystems, Foster City, CA, USA).

### HapMap Analysis

We defined the genomic location for 19386 RefSeq [71] genes from Human Genome Build release 34 and used HapMap phase 1 release data to calculate EHH and iEHH values for each SNP allele within the boundaries of each gene, allowing each allele to decay to an EHH value of at least 0.05, before obtaining the Raw and Rab statistics. We calculated the Rab statistic for each non-African population vs. Africans, as well as for Han Chinese vs. Europeans and Japanese vs. Europeans. As with the SeattleSNPs data, we binned each Rab value by the frequency of the allele in the population in the numerator, calculated the 95% percentile of each bin, and determined the proportion of alleles found above the 95% percentile for each gene. The p-values were determined by comparing the proportion of significant alleles within the gene of interest vs. all other genes in the HapMap Phase I data set. Only genes with at least four Rab or four Raw values were considered, to avoid spurious results caused by too few SNPs.

### Construction of mammalian expression vectors

The open reading frame of the human *TRPV6* ortholog was cloned from a human European placenta and the cDNA sequence verified by sequencing. The ancestral form (157R, 378V, 681T) was generated from the derived form by overlap PCR. Both the derived (CMM) and the ancestral (RVT) forms were ligated into a customized vector (pcDNA3-CFP) that includes the cyan fluorescent protein (CFP) coding sequence 3′ to the multiple cloning sites, to obtain C-terminally CFP-tagged proteins. This construct revealed similar currents as obtained previously with a bicistronic expression vector, pCINeo/IRES-GFP/h*TRPV6*, which was used to co-express human *TRPV6* and enhanced green fluorescent protein (GFP) [72].

### Cell culture and transfection

Human embryonic kidney cells (HEK293) were grown in DMEM containing 10% (v/v) human serum, 2 mM L-glutamine, 2 U/ml penicillin and 2 mg/ml streptomycin at 37°C in a humidity-controlled incubator with 10% CO_2_. HEK293 cells were transiently transfected with the vector constructs. Transfected cells were visually identified in the patch clamp apparatus by their CFP fluorescence.

### Electrophysiology

Electrophysiological methods used here were described previously in detail [38]. Whole-cell currents were measured with an EPC-9 (HEKA Elektronik, Lambrecht, Germany, sampling rate 0.2 ms, 8-Pole Bessel filter 10 kHz) or an L/M-EPC-7 (List Elektronics, Darmstadt, Germany) using ruptured patches. Electrode resistances were between 2 and 5 MΩ, capacitance and access resistances were monitored continuously, and series resistance was maximally compensated. The ramp protocol consisted of linear voltage ramps changing from −150 mV to +100 mV within 400 ms, applied every 5 s from a holding potential of 0 mV. The step protocol consisted of a series of 400 ms long voltage steps applied from a holding potential of +70 mV to −100 mV. Current densities, expressed per unit membrane capacitance, were measured from the current at −100 mV during the ramp protocols.

### Solutions and experimental procedures

The standard extracellular solution (“Krebs”) contained (in mM) 150 NaCl, 6 CsCl, 1.5 CaCl_2_, 1 MgCl_2_, 10 HEPES and 10 glucose, pH 7.4 with CsOH. Nominally free Ca^2+^ solutions were buffered by 5 mM EGTA at a free [Ca^2+^] below 1 nM, as calculated by the CaBuf program (ftp://ftp.cc.kuleuven.be/pub/droogmans/cabuf.zip). The standard internal (pipette) solution contained (in mM) 40 CsCl, 100 Cs-aspartate, 1 MgCl_2_, 10 BAPTA, 4 Na_2_ATP, 10 HEPES, pH 7.2 with CsOH. Cells were kept in a nominally Ca^2+^-free medium to prevent Ca^2+^ overload and exposed for maximal 5 min to a Krebs solution containing 1.5 mM Ca^2+^ before sealing the patch pipette to the cell. If Ca^2+^ (Ba^2+^) was used as the only charge carrier for inward currents, 30mM CaCl_2_ (BaCl_2_) was used in the pipette; all other cations were substituted by NMDG-Cl. All experiments were performed at room temperature (20–22°C).

### Statistical analysis

In all protein function experiments the data are expressed as mean±SEM. Overall statistical significance was determined by analysis of variance (ANOVA, significance level p<0.05).

## Supporting Information

Figure S1 A scatter plot of molecular Fst and lnRHap values for each of the 221 genes from the SeattleSNPs data set. Genes from the candidate region of chromosome 7q34, including the TRPV6 locus, are identified in the plot.
(0.84 MB TIF)

Figure S2 Visual haplotype graph and network of the SeattleSNPs sequence data for the TRPV6 locus. A) A visual haplotype graph of the TRPV6 locus. Each horizontal line is an individual haplotype from a given individual, and each vertical column is a SNP marked by the position of that SNP in the sequence. The major alleles are in blue, while minor alleles are in yellow. B) A MJ network for the European and African SeattleSNP sequence data, with a box around the star-like portion of the network. Branch lengths for the haplotype nodes inside the box were artificially lengthened for illustration purposes.
(3.08 MB TIF)

Figure S3 A density plot of the time since fixation for the putative allele in Europeans, with estimations are based on twelve and ten segregating sites. The 2.5% and 97.5% credible intervals are plotted for both time estimations. The average mode values is ∼7,000 ybp.
(1.12 MB TIF)

Figure S4 A topology graph of the TRPV6 protein illustrating the location of the three non-synonymous mutations, C157R, M378R and M681T and their relationship with putative important functional areas.
(1.42 MB TIF)

Figure S5 Haplotype frequencies of three non-synonymous polymorphisms used as tagging SNPs (rs4987657 (C157R), rs4987667 (M378V) and rs4987682 (M681T)) and genotyped in the CEPH Human Diversity panel and additionally in 31 Ethiopians, 51 Germans, 11 South African Bantu-speakers, and three hunter-gatherer groups from India (44 Koragas, 16 Mullukurunan, and 2 Mullukurumba). The key for inferred haplotypes is in the bottom right corner, with CGC corresponding to the ancestral haplotype and TAT to the derived haplotype.
(4.14 MB TIF)

Figure S6 An overview of the genomic region encompassing the TRPV6/TRPV5 region used to sequence eight blocks in the Karitiana, Han Chinese, highland Papua New Guineans, and the Pathan, previously sequenced in Europeans and African-Americans. The illustration is from the UCSC Genome Browser (chr7:142,278,427-142,330,351) and with the inclusion of a custom track.
(0.83 MB TIF)

Figure S7 Visual haplotype graph of the TRPV5 locus from the SeattleSNPs data set. Each horizontal line is a haplotype and each vertical column is a SNP marked by its position in the TRPV5 sequence. Major alleles are marked in blue, and minor alleles are in yellow. All genotypes were used to infer the haplotypes.
(3.64 MB TIF)

Text S1 Supplementary Results and Discussion, and Materials and Methods
(0.11 MB PDF)

Table S1 SeattleSNPs Genome Scan Statistics
(0.04 MB DOC)

Table S2 Summary Statistics for EPHB6, TRPV6, TRPV5 and KEL
(0.05 MB DOC)

Table S3 World Haplotype Frequencies for C157R, M378V and M681T
(0.03 MB DOC)

Table S4 PCR primers for MALDI-TOF assay
(0.03 MB DOC)

Table S5 Table of PCR and sequencing primers
(0.03 MB DOC)
