# Supplementary figures and images for: Parallel Selection on TRPV6 in Human Populations
Source: PLoS One. 2008 Feb 27;3(2):e1686. doi: 10.1371/journal.pone.0001686 (PMC2246018; doi:10.1371/journal.pone.0001686)

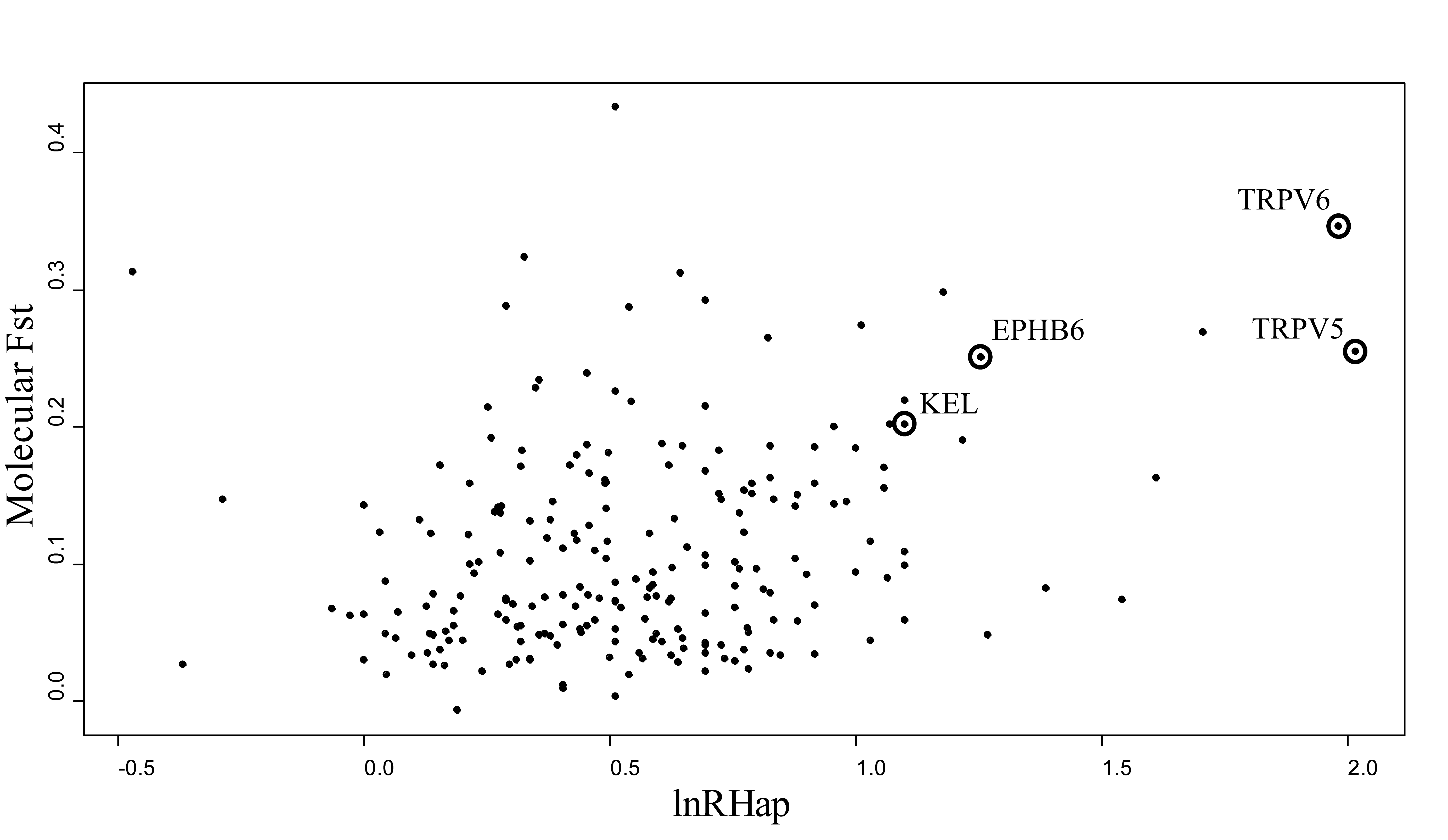

Supplement: Figure S1 — A scatter plot of molecular Fst and lnRHap values for each of the 221 genes from the SeattleSNPs data set. Genes from the candidate region of chromosome 7q34, including the TRPV6 locus, are identified in the plot. (0.84 MB TIF) [file pone.0001686.s001.tif]

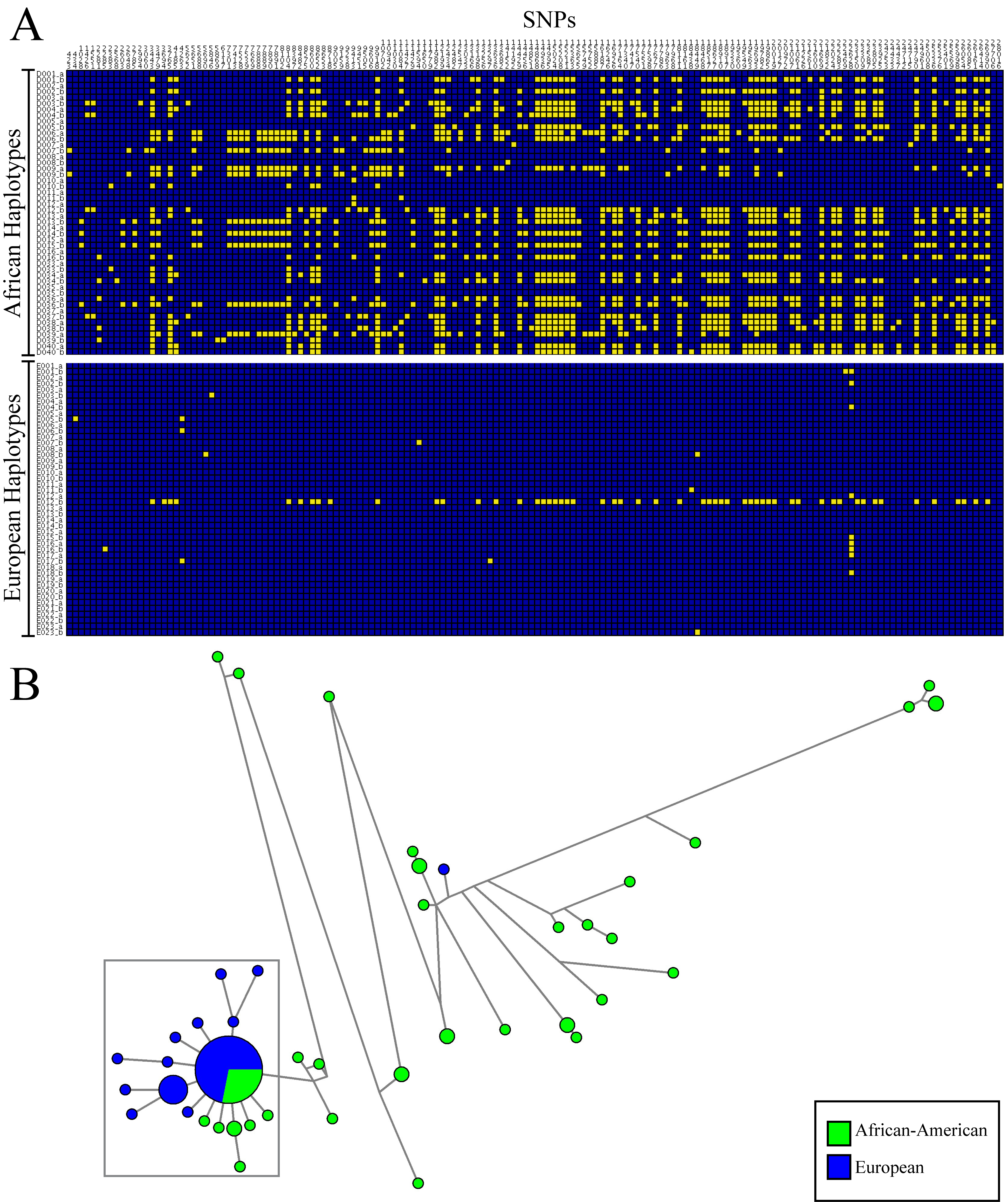

Supplement: Figure S2 — Visual haplotype graph and network of the SeattleSNPs sequence data for the TRPV6 locus. A) A visual haplotype graph of the TRPV6 locus. Each horizontal line is an individual haplotype from a given individual, and each vertical column is a SNP marked by the position of that SNP in the sequence. The major alleles are in blue, while minor alleles are in yellow. B) A MJ network for the European and African SeattleSNP sequence data, with a box around the star-like portion of the network. Branch lengths for the haplotype nodes inside the box were artificially lengthened for illustration purposes. (3.08 MB TIF) [file pone.0001686.s002.tif]

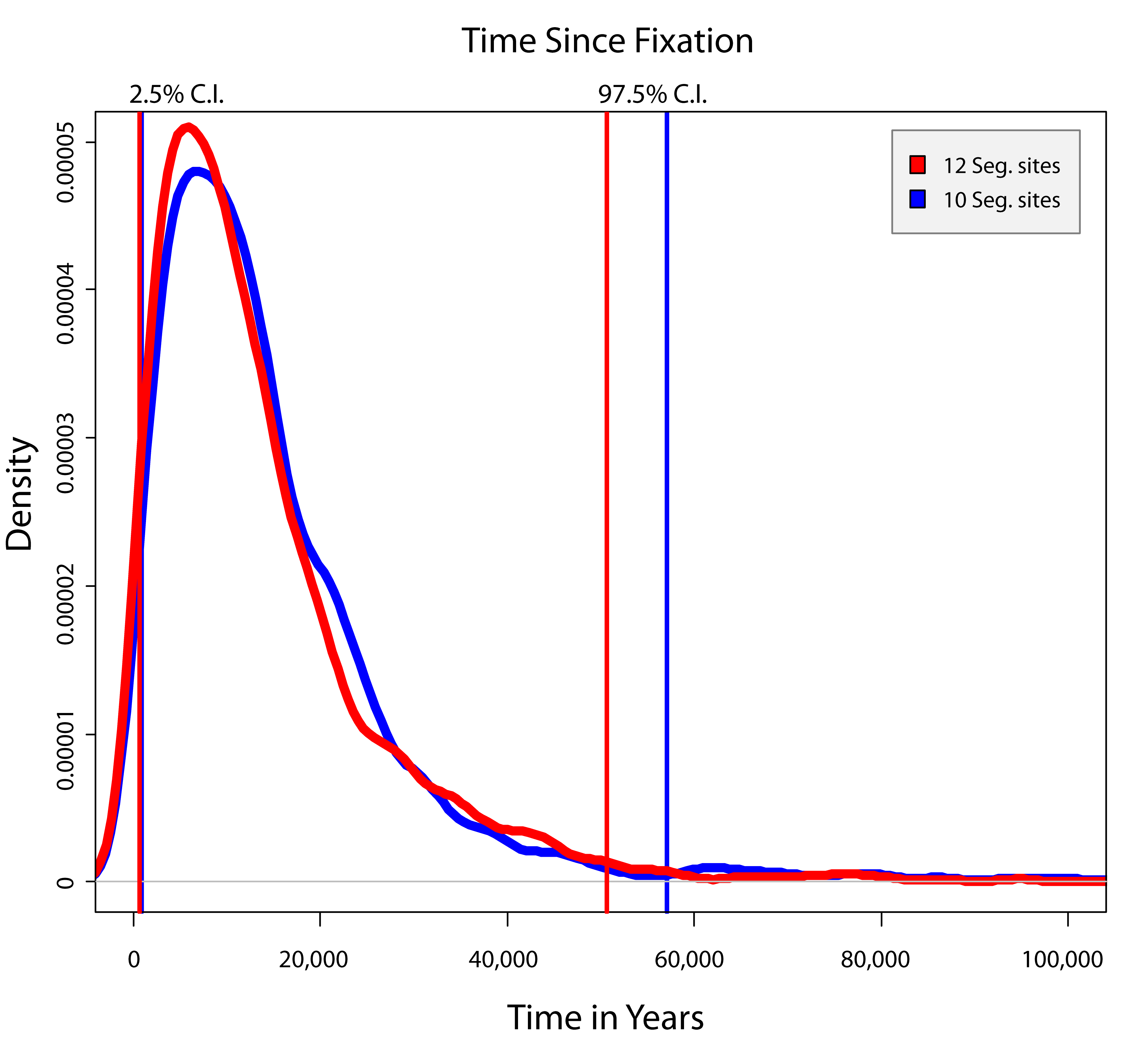

Supplement: Figure S3 — A density plot of the time since fixation for the putative allele in Europeans, with estimations are based on twelve and ten segregating sites. The 2.5% and 97.5% credible intervals are plotted for both time estimations. The average mode values is ∼7,000 ybp. (1.12 MB TIF) [file pone.0001686.s003.tif]

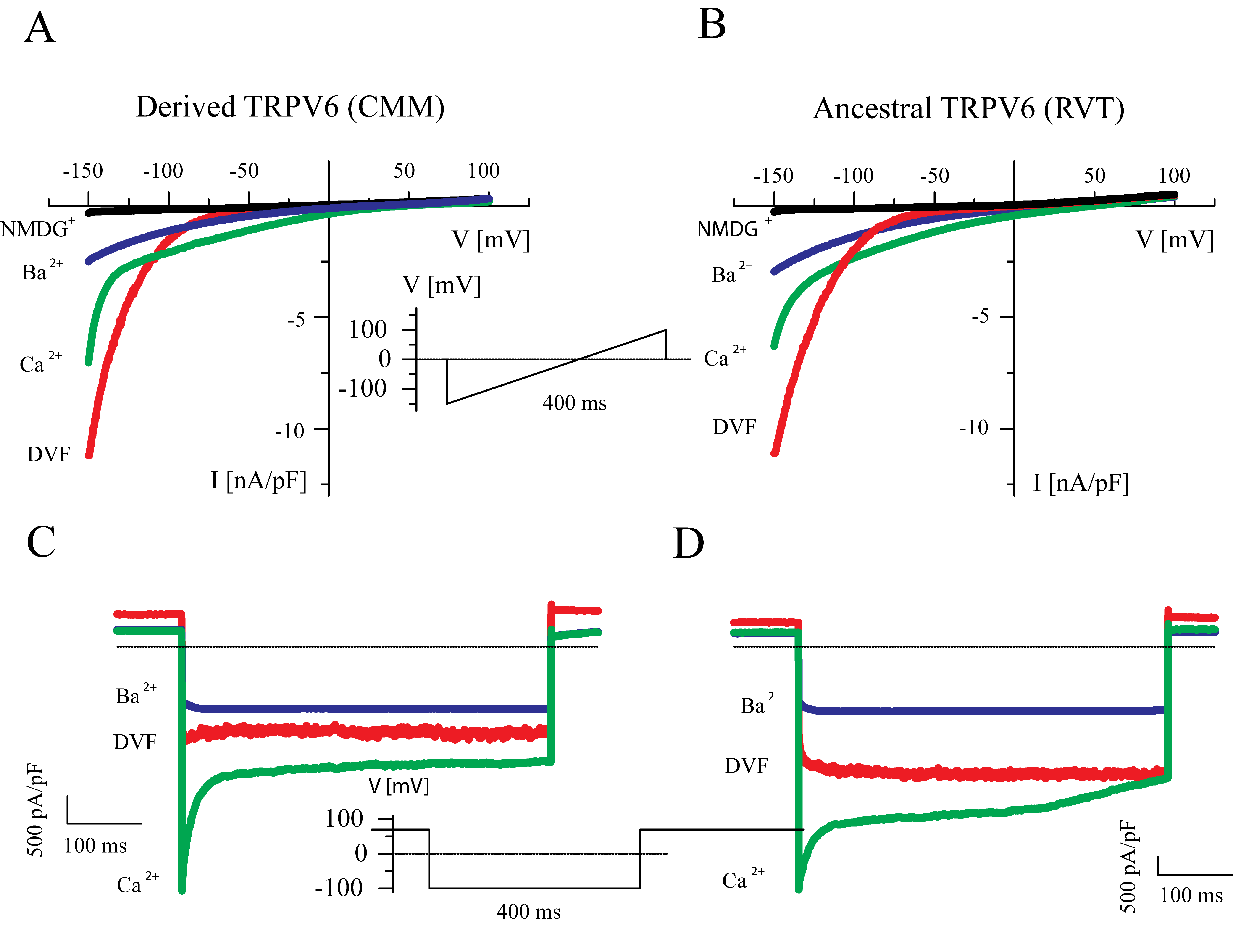

Supplement: Figure S4 — A topology graph of the TRPV6 protein illustrating the location of the three non-synonymous mutations, C157R, M378R and M681T and their relationship with putative important functional areas. (1.42 MB TIF) [file pone.0001686.s004.tif]

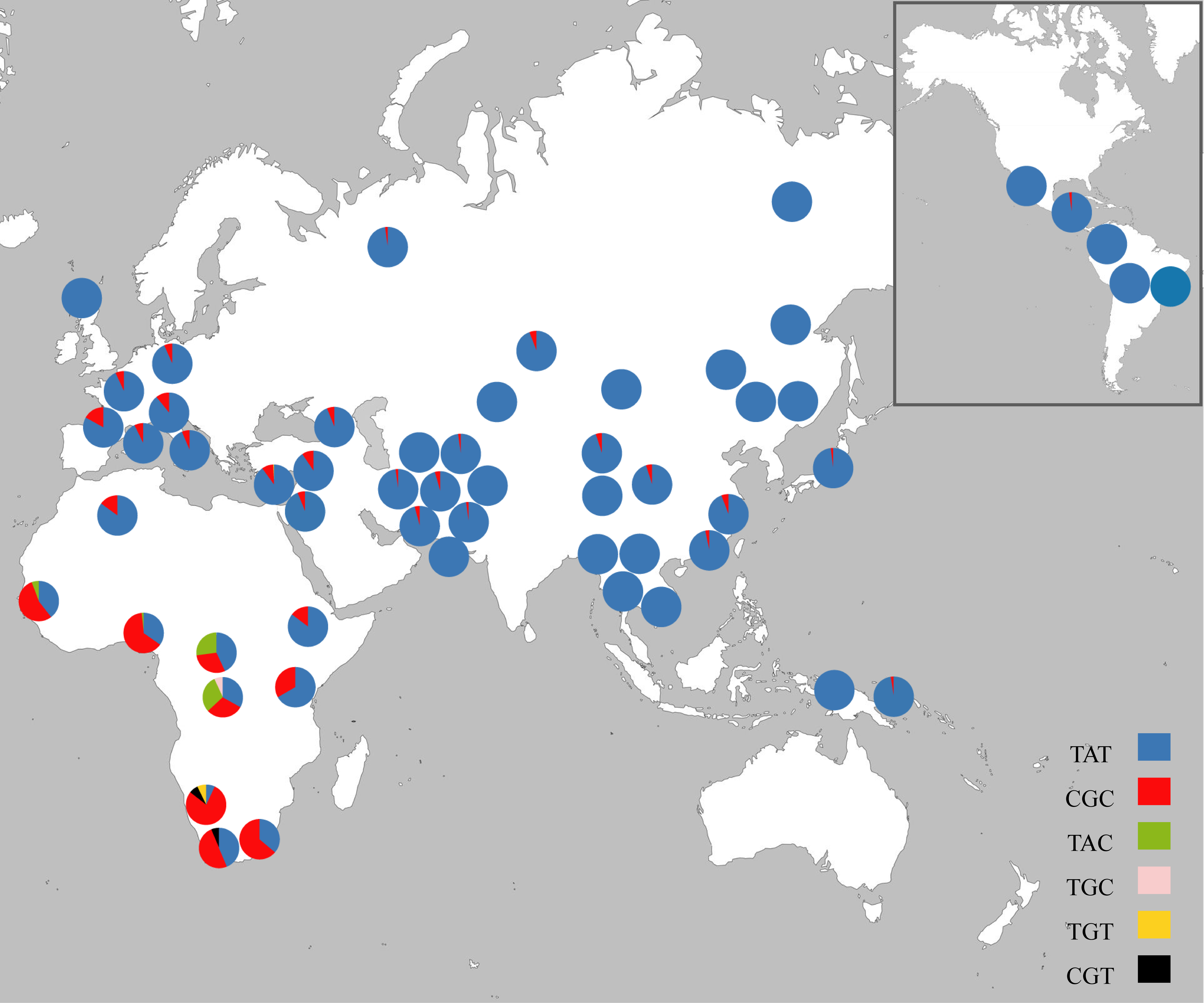

Supplement: Figure S5 — Haplotype frequencies of three non-synonymous polymorphisms used as tagging SNPs (rs4987657 (C157R), rs4987667 (M378V) and rs4987682 (M681T)) and genotyped in the CEPH Human Diversity panel and additionally in 31 Ethiopians, 51 Germans, 11 South African Bantu-speakers, and three hunter-gatherer groups from India (44 Koragas, 16 Mullukurunan, and 2 Mullukurumba). The key for inferred haplotypes is in the bottom right corner, with CGC corresponding to the ancestral haplotype and TAT to the derived haplotype. (4.14 MB TIF) [file pone.0001686.s005.tif]

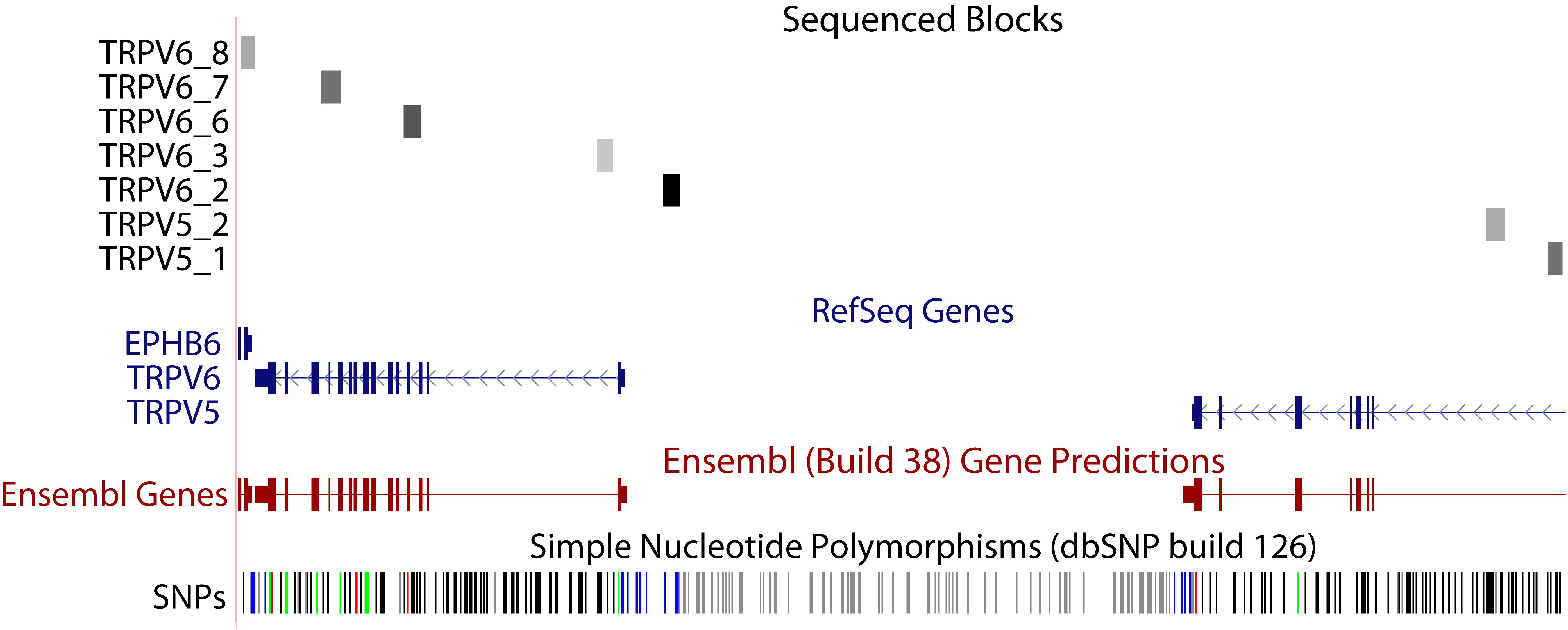

Supplement: Figure S6 — An overview of the genomic region encompassing the TRPV6/TRPV5 region used to sequence eight blocks in the Karitiana, Han Chinese, highland Papua New Guineans, and the Pathan, previously sequenced in Europeans and African-Americans. The illustration is from the UCSC Genome Browser (chr7:142,278,427-142,330,351) and with the inclusion of a custom track. (0.83 MB TIF) [file pone.0001686.s006.tif]

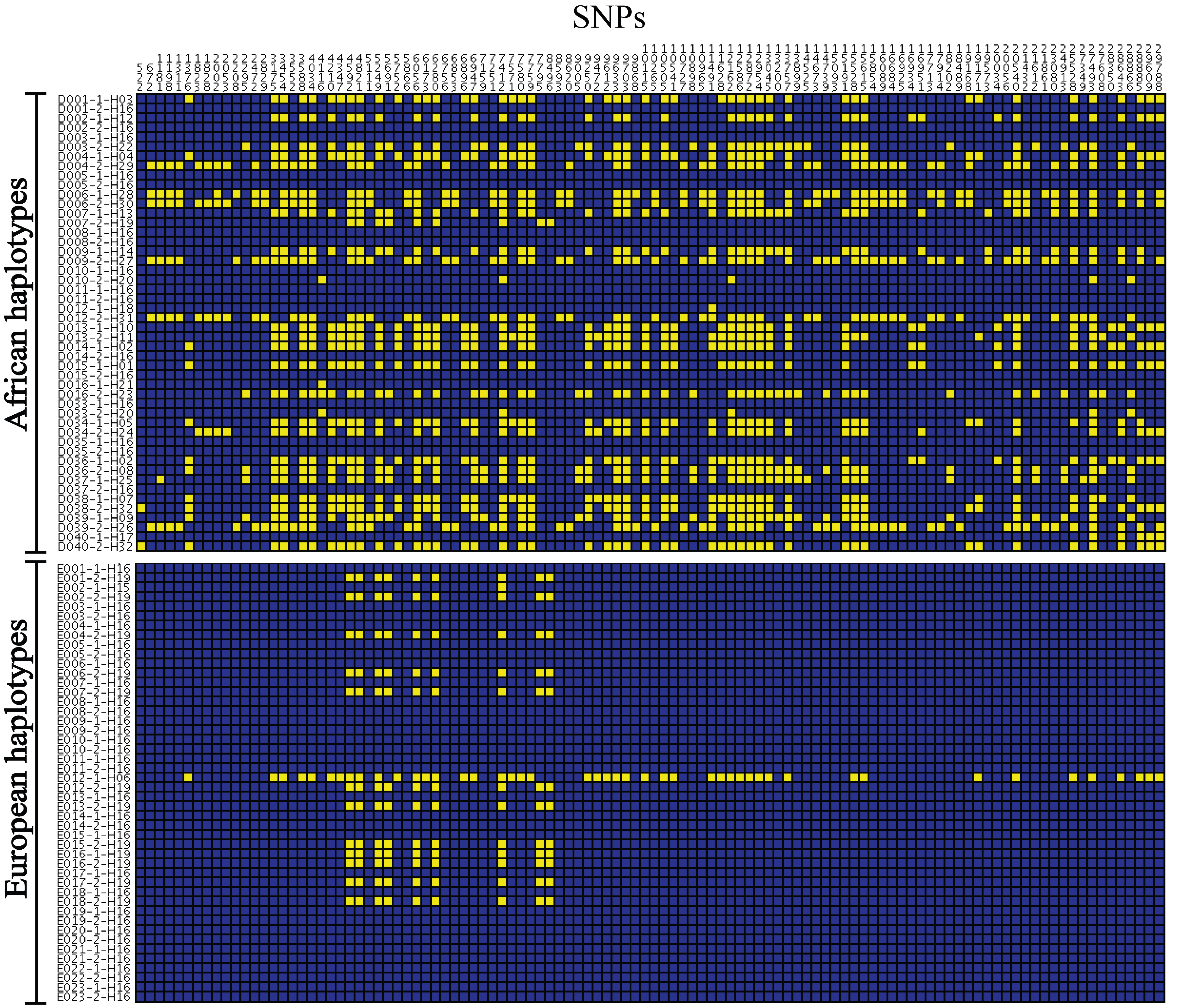

Supplement: Figure S7 — Visual haplotype graph of the TRPV5 locus from the SeattleSNPs data set. Each horizontal line is a haplotype and each vertical column is a SNP marked by its position in the TRPV5 sequence. Major alleles are marked in blue, and minor alleles are in yellow. All genotypes were used to infer the haplotypes. (3.64 MB TIF) [file pone.0001686.s007.tif]
