# Supplementary material for: Parallel Selection on TRPV6 in Human Populations
Source: PLoS One. 2008 Feb 27;3(2):e1686. doi: 10.1371/journal.pone.0001686 (PMC2246018; doi:10.1371/journal.pone.0001686)
Supplement: Text S1 — Supplementary Results and Discussion, and Materials and Methods (0.11 MB PDF) [file pone.0001686.s008.pdf]

**Supplementary Results and Discussion:**

**Genome scan approach identifies *TRPV6* as a candidate for local selection in Europeans**

To identify putative candidate genes for local selection, we calculated two summary statistics, molecular Fst and lnRHap [1, 2], for every gene in the Seattle SNPs data set [3] (Table S1). Molecular Fst [2] is Fst averaged across all polymorphic SNPs within a locus and estimates the amount of genetic differentiation between two or more populations; candidate genes for local selection are expected to have unusually high Fst values [4-7]. lnRHap is a new statistic introduced here, analogous to lnRV and lnRH statistics [1, 8], and is defined for each locus as the natural log of the ratio of the number of haplotypes in the European population to the number of haplotypes in the African-American population. Selection occurring in one population will reduce the number of haplotypes in that population at the selected locus, relative to the number of haplotypes in the population not subject to local selection, resulting in an unusually large, or small, lnRHap value. This analysis identifies *TRPV6* as a potential candidate gene for local selection, because it has both unusually large molecular Fst (0.346) and lnRHap (1.98) values. However, three other genes (*EPHB6*, *TRPV5* and *KEL*) linked to *TRPV6* also exhibit large Fst and lnRHap values (Figure S1). These four genes have also been previously identified as candidate genes for selection through allele frequency spectrum analysis, and for exhibiting departures from neutrality under a variety of demographic models [9, 10].

**TRPV6 Diversity in Europeans**

There is a dramatic and significant paucity of diversity (Table S2) at the *TRPV6* locus in Europeans. Specifically, in the 46 European chromosomes there is one haplotype which

contributes 50 singletons (Figure S2A). This haplotype is closely related to haplotypes in the African (Figure S2B) sample and hence may reflect recent African admixture in this individual, an incomplete sweep, or recombination of the putative advantageous site onto an alternative haplotype. With the exclusion of this chromosome there are only 10 or 12 polymorphic sites ($\pi$ = 0.00003) in *TRPV6* for the European sample (two singletons are present in the European individual with the putative African haplotype and hence cannot be assigned to either haplotype). Of the 10-12 polymorphic sites at *TRPV6* in the European sample, 9-11 are unique to Europeans, while the remaining site is found once in both the European and African samples as a C to T transition at a CpG site and is thus potentially a recurrent mutation. This amount of variation is in stark contrast to the 135 polymorphic sites ($\pi$ = 0.00131) found in African-Americans (Table S2).

**Frequency Spectrum and HKA tests:**

To determine which of the four closely-linked candidate genes (*EPHB6*, *TRPV5, TRPV6,* or *KEL)* might be the target of selection, we employed several tests for departure from neutrality, each of which exploit different aspects of the data: Tajima's D, Fu and Li's D*, Fu and Li's F*, Fay and Wu's H, and the HKA test (Table S1). Each of the four genes exhibit significant departures from neutrality in Europeans for at least four of the five tests. By far the largest departures from neutrality for all five tests in Table S1 are for *TRPV6*. However, the single ancestral chromosome which remains in the Europeans (Figure S2A) is inflating the values for each of the site frequency tests. If we remove this chromosome from the analysis, then *TRPV6* does not remain the most striking candidate of the four neighboring genes for all of the site

frequency tests (Table S1); *EPHB6* shows more significant deviations for some of the tests. This observation is consistent with power analyses of hard and soft sweeps, indicating that site frequency tests have greater power in flanking regions of a selected site starting from the time of fixation [11]. The HKA test generally exhibits the largest departure from neutrality, whether fixation is assumed or not. The results in Figure S1 and Table S1 suggest that *TRPV6* overall exhibits the largest departures from neutrality and hence is the best candidate for having been subject to local selection; therefore we focused further attention on *TRPV6*.

**Age of the putative selection event**

To determine the age of the selective event in Europeans two methods were used. These methods assume that the selective sweep has gone to fixation, so the single non-derived haplotype was removed from the European data. Each of these two methods estimates the time since fixation by essentially determining the time required to accumulate the observed amount of variation on the selected allele. The first method simulates a posterior distribution of the time since fixation, given three summary statistics describing the observed amount of variation, S, the number of segregating sites, D, Tajima's D and H, the number of haplotypes [12]. We performed the analysis assuming the presence of both 10 and 12 segregating sites. The mode was taken as the best estimation of the time since fixation, as recommended by the author [12]. For S = 10 (D = -1.83, H = 10) and S =12 (D = -1.98, H = 11) the mode was approximately 6,000 and 7,000 years before present, respectively. The 95% credible intervals are 708 to 50,600 ybp and 904 to 57,100 ybp for 10 and 12 segregating sites, respectively (see density distribution plot, Figure S3). Second, the method of Slatkin and Hudson [13] assumes a star shaped phylogeny, which appears

to be the case for the European *TRPV6* data (Figure S2B), and estimates the time to the most recent common ancestor based on the observed number of segregating sites, the length of the sequenced region, the number of chromosome and the mutation rate. The time since selection in Europeans was estimated to be 6,835 ybp and 8,200 ybp with 10 and 12 polymorphic sites, respectively. For a single point estimation the average over all results was taken resulting in an estimated time since fixation of 7,000 years before present, comparable to a previous estimate of 10,000 ybp [9] obtained by yet another method.

**Worldwide Genotyping of TRPV6**

The relatively recent date for the putative selection on TRPV6 in Europeans, coupled with the fact that TRPV6 is involved in dietary calcium uptake, suggested that the putative selection on TRPV6 may be related to dairying [9]. To test this hypothesis, we investigated the worldwide distribution of the putatively-selected haplotype by genotyping three *TRPV6* tagging SNPs for the selected haplotype in the CEPH diversity panel [14] and additionally in samples of Ethiopians, Germans, South African Bantu-speakers, and three hunter-gatherer groups from India. These three tagging SNPs are non-synonymous polymorphisms (C157R, M378V, M681T; Figure S4) and they are the only non-synonymous polymorphisms in the SeattleSNPs data for this locus. They each occur in the SeattleSNPs data in the derived state at 98% frequency in Europeans, and at 52%, 52%, and 50% frequency respectively in African-Americans. These three amino acid substitutions are an obvious candidate for a functional difference influencing *TRPV6* and thus for defining the putatively selected haplotype. Moreover, a recent branch-sites analysis [15] of the protein coding sequence of *TRPV6* from human, mouse, rat and chimpanzee

indicated that *TRPV6* had experienced accelerated evolution on the human lineage, and that the three amino acid sites C157R, M378V and M691T have a high probability of having evolved under positive selection [16]. Thus, a number of lines of evidence suggest that the derived haplotype for these three non-synonymous SNPs has been the target of local selection in Europeans.

Genotyping of 1206 individuals indicates that the derived haplotype defined by these three sites is fixed (or nearly so) in all non-African populations, a nd is found at lower frequencies in all African populations (Figure S5). These results agree with another recent study [16]; haplotype frequencies for each population can be found in Table S3.

## Supplementary Materials and Methods:

### Genome Scan Summary Statistics

Our scan for candidate genes of local directional selection is based on two summary statistics: molecular Fst values and lnRHap values [1, 2]. Molecular Fst, or Weir and Cockerham's $\theta_W$, was calculated with Fstat 2.9.3.2, while lnRHap is defined for each locus as the natural log of the ratio of the number of haplotypes in the European population to the number of haplotypes in the African population. The number of haplotypes in each population was determined from PHASE v2.0 [17, 18] output files from the SeattleSNPs website. These two summary statistics were then plotted in a scatter plot to identify outliers, i.e. those loci with unusually large molecular Fst values and/or large or small lnRHap values.

### Neutrality Tests

The program DnaSP 4.0 [19] was used to calculate additional summary statistics and perform tests for departures from neutrality. The statistical significance of $\pi$ values, Tajima's D and Fay and Wu's H values were estimated by simulation using the observed number of segregating sites, no recombination and a demographic model previously found to fit the observed pattern of genetic variation in human populations [20], described below. Statistical significance of Fu and Li's D* and F* tests and the HKA test were determined by DnaSP using published critical values [21], and a chi-square distribution with one degree of freedom, respectively. We inferred haplotypes for *TRPV6* and neighboring genes with Phase v2.0.2 [17, 18], using all sites from the genotype files downloaded from the SeattleSNPs website. Phase was inferred here because SeattleSNPs phase files had SNPs below a frequency of 5% removed prior to haplotype inference. Complete haplotype sequences for each individual were then created using reference sequences submitted by SeattleSNPs (AY280502, AY225461, AY206695, AY228336, and AF539592 for *EPHB6*, *TRPV6*, *TRPV5*, *KEL*, and PON1, respectively) and the inferred phase. To retrieve the orthologous *Pan troglodytes* sequence, each reference sequence was subjected to a Blat search against the Chimp January 2006 [22] assembly using the UCSC browser. The retrieved *Pan troglodytes* sequence and one human sequence, created with phased haplotype data and the reference sequence, were aligned with T-coffee [23] and all other sequences were then aligned by hand following the T-coffee alignment using MEGA 3.1 [24]. For the HKA test, which compares the diversity within populations and the divergence between humans and chimpanzee at the putatively selected locus and a putative neutral locus [25], data were also obtained from the SeattleSNPs website for the PON1 gene, located on the same chromosome as *TRPV6* but 47.5 mega-bases away, to serve as the neutral locus in the test.

**CEPH Diversity Panel Genotyping**

Three tagging SNPs in the *TRPV6* gene were genotyped in the CEPH diversity panel [14] and additionally in 31 Ethiopians, 51 Germans, 11 South African Bantu-speakers, and three hunter-gatherer groups from India (44 Koragas, 16 Mullukurunan, and 2 Mullukurumba). The tagging SNPs are three non-synonymous polymorphisms: rs4987657, C157R; rs4987667, M378V; and rs4987682, M681T. These three non-synonymous mutations were genotyped by a single base pair extension (SBE) multiplex reaction (Table S4) on an Autoflex MALDI-TOF mass spectrometer (Bruker Daltonics GmbH, Bremen, Germany). Design of the single base extension MALDI-TOF primers was accomplished with an early un-released version of CalcDalton [26]. Subsequent to the SBE reaction multiplex, the amplified templates were purified by SAP/ExoI digestion in a 10µl reaction containing 8µl of template DNA, 0.3 U Shrimp alkaline phosphatase (SAP) (Amersham, Buckinghamshire, England) and 0.2 U Exonuclease I (ExoI) (NEB, Ipswich, MA, USA). Digestions took place at 37° for 60 min. SBE reactions included the 10 µl of purified PCR template, 6.25mM $MgCl_2$ (Solis Biodyne, Tartu, Estonia), 125µM ddNTPs (Carl Roth, Karlsruhe, Germany), 312.5nM of each extension primer (Biotez, Berlin, Germany), and 1µl of Thermipol polymerase (Solis Biodyne, Tartu, Estonia). SBE reactions were carried out in GeneAmp 9600 thermocyclers (Perkin Elmer, Wellesley, MA, USA) and consisted of an initial denaturation step at 94° for 4 min, then 34 cycles of denaturation at 94° for 10 sec, annealing at 55° for 30 sec and extension for 10 sec at 72°. The SBE reaction products were purified with the Genostrep 96 Kit (Bruker Daltonics, Bremen, Germany) following the manufacturer's protocol. Measurements from each purified sample were replicated four times, with each measurement

consisting of the average of five spectra, each of which was derived from 50 laser shots. Results were analyzed with the software FlexAnalysis version 2.0 (Bruker Daltonics GmbH, Bremen, Germany)

**Dating the putative selection event**

Two methods were used to determine the age of the putative selective event in Europeans at the *TRPV6* locus using the SeattleSNP data. First, a posterior distribution of time since selection was simulated based on observed diversity levels at the putatively selected locus. This was done using the program msHH [12], which utilizes three summary statistics of the data (the number of segregating sites (S), the number of haplotypes (H), and Tajima's D (D)). From the distributions we calculated the 95% credible interval or C.I. and took the mode as the best estimates (Figure S3). Second, we used a method which estimates the time to the most recent common ancestor for a locus given that it has a star shaped phylogeny [13]. T, the time since the common ancestor, is estimated as:

$$T_{\text{gen}} = \frac{S}{nt \cdot N \cdot \mu}$$

where $T_{gen}$ is time in generations, S is the number of segregating sites, nt is the number of sequenced nucleotides, N is the number of chromosomes and μ is the mutation rate [27, 28]. For these analyses we used phased data that we produced by including all sites from the SeattleSNPs data. In both situations we assumed that the selective sweep has reached fixation and thus we removed the single ancestral African chromosome from the European data, prior to dating. We

used a generation time of 20 years, a mutation rate ($\mu$) of 2.3 x $10^{-8}$ [29, 30], and a rho ($\rho$) of 0.001 [30, 31].

**Supplementary References:**


1. Schlotterer, C., *A microsatellite-based multilocus screen for the identification of local selective sweeps.* Genetics, 2002. **160**(2): p. 753-63.
2. Weir, B.S. and C.C. Cockerham, *Estimating F-Statistics for the Analysis of Population Structure.* Evolution, 1984. **38**(6): p. 1358-1370.
3. SeattleSNPs, *NHLBI Program for Genomic Applications, SeattleSNPs, Seattle, WA (URL: http://pga.gs.washington.edu).*
4. Akey, J.M., et al., *Interrogating a high-density SNP map for signatures of natural selection.* Genome Res, 2002. **12**(12): p. 1805-14.
5. Bamshad, M. and S.P. Wooding, *Signatures of natural selection in the human genome.* Nat Rev Genet, 2003. **4**(2): p. 99-111.
6. Beaumont, M.A., *Adaptation and speciation: what can Fst tell us?* Trends in Ecology & Evolution, 2005. **20**(8): p. 435.
7. Beaumont, M.A. and D.J. Balding, *Identifying adaptive genetic divergence among populations from genome scans.* Mol Ecol, 2004. **13**(4): p. 969-80.
8. Schlotterer, C., M. Kauer, and D. Dieringer, *Allele excess at neutrally evolving microsatellites and the implications for tests of neutrality.* Proc Biol Sci, 2004. **271**(1541): p. 869-74.
9. Akey, J.M., et al., *Population History and Natural Selection Shape Patterns of Genetic Variation in 132 Genes.* PLoS Biol, 2004. **2**(10): p. E286.
10. Stajich, J.E. and M.W. Hahn, *Disentangling the Effects of Demography and Selection in Human History.* Mol Biol Evol, 2004.
11. Pennings, P.S. and J. Hermisson, *Soft Sweeps III: The Signature of Positive Selection from Recurrent Mutation.* PLoS Genet, 2006. **2**(12): p. e186.
12. Przeworski, M., *Estimating the time since the fixation of a beneficial allele.* Genetics, 2003. **164**(4): p. 1667-76.
13. Slatkin, M. and R.R. Hudson, *Pairwise comparisons of mitochondrial DNA sequences in stable and exponentially growing populations.* Genetics, 1991. **129**(2): p. 555-62.
14. Cann, H.M., et al., *A human genome diversity cell line panel.* Science, 2002. **296**(5566): p. 261-2.
15. Zhang, J., R. Nielsen, and Z. Yang, *Evaluation of an improved branch-site likelihood method for detecting positive selection at the molecular level.* Mol Biol Evol, 2005. **22**(12): p. 2472-9.
16. Akey, J.M., et al., *TRPV6 exhibits unusual patterns of polymorphism and divergence in worldwide populations.* Hum Mol Genet, 2006. **15**(13): p. 2106-13.
17. Stephens, M. and P. Scheet, *Accounting for decay of linkage disequilibrium in haplotype inference and missing-data imputation.* Am J Hum Genet, 2005. **76**(3): p. 449-62.
18. Stephens, M., N.J. Smith, and P. Donnelly, *A new statistical method for haplotype reconstruction from population data.* Am J Hum Genet, 2001. **68**(4): p. 978-89.
19. Rozas, J., et al., *DnaSP, DNA polymorphism analyses by the coalescent and other methods.* Bioinformatics, 2003. **19**(18): p. 2496-7.
20. Schaffner, S.F., et al., *Calibrating a coalescent simulation of human genome sequence variation.* Genome Res, 2005. **15**(11): p. 1576-83.

21. Fu, Y.X. and W.H. Li, *Statistical tests of neutrality of mutations.* Genetics, 1993. **133**(3): p. 693-709.
22. The Chimpanzee Sequencing and Analysis Consortium, *Initial sequence of the chimpanzee genome and comparison with the human genome.* Nature, 2005. **437**(7055): p. 69.
23. Notredame, C., D.G. Higgins, and J. Heringa, *T-Coffee: A novel method for fast and accurate multiple sequence alignment.* J Mol Biol, 2000. **302**(1): p. 205-17.
24. Kumar, S., K. Tamura, and M. Nei, *MEGA3: Integrated software for Molecular Evolutionary Genetics Analysis and sequence alignment.* Brief Bioinform, 2004. **5**(2): p. 150-63.
25. Hudson, R.R., M. Kreitman, and M. Aguade, *A test of neutral molecular evolution based on nucleotide data.* Genetics, 1987. **116**(1): p. 153-9.
26. Kirsten, H., et al., *CalcDalton: a tool for multiplex genotyping primer design for single-base extension reactions using cleavable primers.* Biotechniques, 2006. **40**(2): p. 158, 160, 162.
27. Baudry, E., B. Viginier, and M. Veuille, *Non-African populations of Drosophila melanogaster have a unique origin.* Mol Biol Evol, 2004. **21**(8): p. 1482-91.
28. Haddrill, P.R., et al., *Multilocus patterns of nucleotide variability and the demographic and selection history of Drosophila melanogaster populations.* Genome Res, 2005. **15**(6): p. 790-9.
29. Fischer, A., et al., *Evidence for a Complex Demographic History of Chimpanzees.* Mol Biol Evol, 2004. **21**(5): p. 799-808.
30. Frisse, L., et al., *Gene conversion and different population histories may explain the contrast between polymorphism and linkage disequilibrium levels.* Am J Hum Genet, 2001. **69**(4): p. 831-43.
31. Ptak, S.E., K. Voelpel, and M. Przeworski, *Insights into recombination from patterns of linkage disequilibrium in humans.* Genetics, 2004. **167**(1): p. 387-97.

**Figure Legends:**

Figure S1. A scatter plot of molecular Fst and lnRHap values for each of the 221 genes from the SeattleSNPs data set. Genes from the candidate region of chromosome 7q34, including the *TRPV6* locus, are identified in the plot.

Figure S2. Visual haplotype graph and network of the SeattleSNPs sequence data for the *TRPV6* locus. A) A visual haplotype graph of the *TRPV6* locus. Each horizontal line is an individual haplotype from a given individual, and each vertical column is a SNP marked by the position of that SNP in the sequence. The major alleles are in blue, while minor alleles are in yellow. B) A MJ network for the European and African SeattleSNP sequence data, with a box around the star-like portion of the network. Branch lengths for the haplotype nodes inside the box were artificially lengthened for illustration purposes.

Figure S3. A density plot of the time since fixation for the putative allele in Europeans, with estimations are based on twelve and ten segregating sites. The 2.5% and 97.5% credible intervals are plotted for both time estimations. The average mode values is ~7,000ybp.

Figure S4. A topology graph of the *TRPV6* protein illustrating the location of the three non-synonymous mutations, C157R, M378R and M681T and their relationship with putative important functional areas.

Figure S5. Haplotype frequencies of three non-synonymous polymorphisms used as tagging SNPs (rs4987657 (C157R), rs4987667 (M378V) and rs4987682 (M681T)) and genotyped in the CEPH Human Diversity panel and additionally in 31 Ethiopians, 51 Germans, 11 South African Bantu-speakers, and three hunter-gatherer groups from India (44 Koragas, 16 Mullukurunan, and 2 Mullukurumba). The key for inferred haplotypes is in the bottom right corner, with CGC corresponding to the ancestral haplotype and TAT to the derived haplotype.

Figure S6. An overview of the genomic region encompassing the *TRPV6*/*TRPV5* region used to sequence eight blocks in the Karitiana, Han Chinese, highland Papua New Guineans, and the Pathan, previously sequenced in Europeans and African-Americans. The illustration is from the UCSC Genome Browser (chr7:142,278,427-142,330,351) and with the inclusion of a custom track.

Figure S7. Visual haplotype graph of the *TRPV5* locus from the SeattleSNPs data set. Each horizontal line is a haplotype and each vertical column is a SNP marked by its position in the *TRPV5* sequence. Major alleles are marked in blue, and minor alleles are in yellow. All genotypes were used to infer the haplotypes.
