# Supplementary material for: Parallel Selection on TRPV6 in Human Populations
Source: PLoS One. 2008 Feb 27;3(2):e1686. doi: 10.1371/journal.pone.0001686 (PMC2246018; doi:10.1371/journal.pone.0001686)
Supplement: Table S1 — SeattleSNPs Genome Scan Statistics (0.04 MB DOC) [file pone.0001686.s009.doc]

**Table S1: SeattleSNPs Genome Scan Summary Statistics**

**HugoName African_hap European_hap lnRHap mol_Fst**

A4GALT 40 29 0.32 0.18

ABO 41 30 0.31 0.05

ACE2 18 10 0.59 0.09

ACHE 18 11 0.49 0.10

ADRB1 22 12 0.61 0.04

AGTRAP 35 21 0.51 0.09

ALOX12 35 16 0.78 0.02

ALOX15 31 20 0.44 0.08

ALOX5AP 37 24 0.43 0.12

APOH 34 16 0.75 0.07

AQP1 36 26 0.33 0.32

BDKRB2 18 12 0.41 0.01

BF 16 12 0.29 0.08

BGLAP 9 13 -0.37 0.03

BSG 22 14 0.45 0.24

C1QA 13 6 0.77 0.15

C2 21 11 0.65 0.05

C3 43 39 0.10 0.03

C3AR1 29 10 1.06 0.09

CAT 25 14 0.58 0.12

CCR2 14 7 0.69 0.04

CD36 38 26 0.38 0.13

CD9 40 15 0.98 0.15

CEBPB 8 6 0.29 0.29

CHUK 19 7 1.00 0.18

CKLF 20 12 0.51 0.23

CKM 31 25 0.22 0.16

COCH 43 33 0.26 0.14

CPB2 25 15 0.51 0.07

CRF 27 8 1.22 0.19

CRP 10 4 0.92 0.16

CSF2 15 6 0.92 0.07

CSF3 13 7 0.62 0.07

CSF3R 32 13 0.90 0.09

CXCL12 31 15 0.73 0.04

CXCR4 10 5 0.69 0.17

CYP4A11 16 8 0.69 0.22

CYP4F2 25 10 0.92 0.03

CYP4F3 40 26 0.43 0.07

DAF 20 17 0.16 0.03

DCN 23 14 0.50 0.18

DO 14 11 0.24 0.02

*EPHB6* 28 8 1.25 0.25

F10 46 40 0.14 0.05

F11 23 20 0.14 0.08

F12 22 14 0.45 0.19

F13A1 47 45 0.04 0.09

F13B 21 7 1.10 0.22

F2 19 7 1.00 0.09

F2R 28 18 0.44 0.05

F2RL1 19 13 0.38 0.05

F2RL2 28 15 0.62 0.08

F2RL3 22 10 0.79 0.16

F3 28 15 0.62 0.03

F5 47 41 0.14 0.12

F7 23 13 0.57 0.06

F8 18 11 0.49 0.14

F9 27 15 0.59 0.05

FCN3 14 3 1.54 0.07

FGA 11 7 0.45 0.06

FGB 8 7 0.13 0.05

FGG 10 5 0.69 0.10

FGL2 13 5 0.96 0.20

FSBP 8 8 0.00 0.06

FUK 21 11 0.65 0.19

FUT1 14 12 0.15 0.17

FUT2 24 14 0.54 0.29

FUT3 30 19 0.46 0.17

GATA3 32 20 0.47 0.11

GP1BA 13 9 0.37 0.08

GYPC NA NA NA 0.07

HABP2 45 42 0.07 0.06

HMOX1 22 17 0.26 0.19

ICAM1 29 22 0.28 0.11

ICAM4 7 5 0.34 0.10

IFNAR1 25 14 0.58 0.08

IFNAR2 29 24 0.19 -0.01

IFNG 10 8 0.22 0.09

IFNGR1 30 21 0.36 0.05

IFNGR2 26 21 0.21 0.10

IGF1 24 16 0.41 0.01

IGF2 18 6 1.10 0.06

IGF2AS 40 18 0.80 0.10

IKBKB 20 12 0.51 0.43

IL10 11 8 0.32 0.06

IL10RA 15 16 -0.06 0.07

IL10RB 45 37 0.20 0.08

IL11 28 12 0.85 0.03

IL11RA 17 8 0.75 0.10

IL12A 16 7 0.83 0.16

IL12B 16 7 0.83 0.08

IL12RB1 40 23 0.55 0.09

IL12RB2 36 22 0.49 0.16

IL13 30 14 0.76 0.14

IL13RA2 13 4 1.18 0.30

IL15RA 38 32 0.17 0.04

IL16 28 17 0.50 0.03

IL17 28 25 0.11 0.13

IL17B 8 6 0.29 0.06

IL17RB 39 15 0.96 0.14

IL19 11 8 0.32 0.04

IL1A 16 7 0.83 0.04

IL1B 27 14 0.66 0.11

IL1F10 30 19 0.46 0.13

IL1F5 14 10 0.34 0.13

IL1F6 23 10 0.83 0.15

IL1F7 11 4 1.01 0.27

IL1F9 11 8 0.32 0.17

IL1R1 39 27 0.37 0.05

IL1R2 35 16 0.78 0.05

IL1RN 30 14 0.76 0.10

IL2 6 8 -0.29 0.15

IL20 21 12 0.56 0.04

IL21 12 8 0.41 0.11

IL21R 46 40 0.14 0.03

IL22 14 10 0.34 0.03

IL22RA2 27 16 0.52 0.07

IL24 14 5 1.03 0.12

IL26 41 25 0.49 0.12

IL2RA 42 37 0.13 0.07

IL2RB 44 36 0.20 0.04

IL2RG 12 5 0.88 0.14

IL3 12 6 0.69 0.04

IL3RA 48 45 0.06 0.05

IL4 35 17 0.72 0.18

IL4R 35 21 0.51 0.07

IL5 11 2 1.70 0.27

IL5RA 48 46 0.04 0.05

IL6 18 14 0.25 0.21

IL7R 20 10 0.69 0.02

IL8 10 2 1.61 0.16

IL8RA 21 7 1.10 0.10

IL8RB 18 9 0.69 0.06

IL9 10 5 0.69 0.04

IL9R 37 24 0.43 0.18

IRAK4 23 8 1.06 0.17

ITGA2 44 42 0.05 0.02

ITGA8 46 35 0.27 0.14

JAK3 45 30 0.41 0.08

*KEL* 36 12 1.10 0.20

KLK1 20 12 0.51 0.04

KLKB1 37 21 0.57 0.03

KNG 43 32 0.30 0.03

LEP 17 12 0.35 0.23

LTA 14 12 0.15 0.04

LTB 5 5 0.00 0.03

LU 36 19 0.64 0.05

MAP3K8 32 14 0.83 0.19

MC1R 10 10 0.00 0.14

MCP 33 29 0.13 0.04

MGP 9 5 0.59 0.09

MMP3 17 8 0.75 0.08

MMP9 16 9 0.58 0.08

NFKBIA 23 20 0.14 0.05

NFKBIB 25 12 0.73 0.03

NFKBIE 10 16 -0.47 0.31

NOS3 33 25 0.28 0.14

PCSK9 47 38 0.21 0.12

PFC 14 5 1.03 0.04

PLAT 41 17 0.88 0.15

PLAU 12 10 0.18 0.06

PLAUR 46 35 0.27 0.06

PLG 36 30 0.18 0.07

PLTP 23 17 0.30 0.07

PON1 41 27 0.42 0.17

PON2 30 22 0.31 0.03

PON3 29 12 0.88 0.06

PPARA 47 25 0.63 0.13

PPARG 32 31 0.03 0.12

PROC 17 8 0.75 0.03

PROCR 9 4 0.81 0.08

PROS1 23 15 0.43 0.12

PROZ 24 6 1.39 0.08

PTGS1 31 18 0.54 0.22

PTGS2 21 13 0.48 0.08

RELA 8 6 0.29 0.07

RIPK1 44 22 0.69 0.29

SCYA2 24 15 0.47 0.06

SELE 22 15 0.38 0.15

SELL 32 16 0.69 0.11

SELP 45 34 0.28 0.14

SELPLG 45 32 0.34 0.07

SEMA7A 25 11 0.82 0.27

SERPINA5 24 19 0.23 0.10

SERPINC1 23 8 1.06 0.16

SERPINE1 23 12 0.65 0.04

SERPING1 10 6 0.51 0.05

SFTPA1 35 17 0.72 0.15

SFTPA2 39 21 0.62 0.17

SFTPB 31 15 0.73 0.15

SFTPC 35 36 -0.03 0.06

SFTPD 31 19 0.49 0.16

SLC14A1 38 20 0.64 0.31

SMP1 29 20 0.37 0.12

STAT3 33 15 0.79 0.15

STAT4 31 19 0.49 0.16

STAT6 24 11 0.78 0.05

TF 38 21 0.59 0.08

TFPI 41 26 0.46 0.08

TGFB3 32 9 1.27 0.05

THBD 12 7 0.54 0.02

THBS4 36 19 0.64 0.03

TIRAP 23 10 0.83 0.06

TNF 10 6 0.51 0.00

TNFAIP1 9 6 0.41 0.06

TNFAIP2 31 20 0.44 0.05

TNFAIP3 13 6 0.77 0.12

TNFRSF1A 32 11 1.07 0.20

TNFRSF1B 46 39 0.17 0.05

TRADD 11 6 0.61 0.19

TRAF2 38 21 0.59 0.05

TRAF6 30 12 0.92 0.19

*TRPV5* 30 4 2.01 0.26

*TRPV6* 29 4 1.98 0.35

TTRAP 28 20 0.34 0.03

TYK2 26 12 0.77 0.04

USF1 10 7 0.36 0.23

VCAM1 43 23 0.63 0.10

VEGF 37 25 0.39 0.04

VKORC1 12 5 0.88 0.10

VTN 12 4 1.10 0.11
