# Supplementary material for: Parallel Selection on TRPV6 in Human Populations
Source: PLoS One. 2008 Feb 27;3(2):e1686. doi: 10.1371/journal.pone.0001686 (PMC2246018; doi:10.1371/journal.pone.0001686)
Supplement: Table S2 — Summary Statistics for EPHB6, TRPV6, TRPV5 and KEL (0.05 MB DOC) [file pone.0001686.s010.doc]

**Table S2: Summary statistics for the genes *EPHB6*, *TRPV6*, *TRPV5*, and *KEL***.

| **Locus** | **Population** | **n** | **S** | **θ_W_** | **π** | **Tajima's D** | **Fu and Li's D*** | **Fu and Li's F*** | **Fay and Wu's H** | **HKA X^2^** |
| --- | --- | --- | --- | --- | --- | --- | --- | --- | --- | --- |
| *EPHB6* | African | 48 | 93 | 20.96 | 16.24 | -0.86 | -0.25 | -0.57 | 2.66 | 1.41 |
|  | European | 46 | 37 | 8.42 | 3.61** | -2.02* | -4.23** | -4.10** | -5.64 | 5.75* |
|  | European[-1] | 45 | 23 | 5.26 | 2.81 | -1.60* | -3.73** | -3.55** | -0.64 | 10.10** |
| *TRPV6* | African | 48 | 135 | 30.42 | 37.04 | 0.79 | 0.9 | 1.02 | 3.85 | 2.81 |
|  | European | 46 | 60 | 13.65 | **3.11***** | **-2.75***** | **-6.15**** | **-5.86**** | **-50.19**** | 7.03** |
|  | European[-1] | 45 | 10 | 2.29 | 0.85* | -1.83* | -2.79* | -2.92* | 0.75 | **24.74***** |
| *TRPV5* | African | 48 | 183 | 41.24 | 35.85 | -0.48 | -0.92 | -0.9 | -19.26 | 0.02 |
|  | European | 46 | 63 | 14.33 | 6.02** | -2.09* | -4.97** | -4.68** | -36.12* | 3.61 |
|  | European[-1] | 45 | 25 | 5.72 | 4.22 | -0.94 | -2.44 | -2.28 | -2.79 | 13.20*** |
| *KEL* | African | 48 | 124 | 27.94 | 23.59 | -0.56 | -0.48 | -0.61 | -5.57 | 0.25 |
|  | European | 46 | 45 | 10.24 | 3.63** | -2.26** | -3.78** | -3.84** | -27.86* | 3.6 |
|  | European[-1] | 45 | 28 | 6.4 | 3.11* | -1.79* | -2.04 | -2.32 | -8.99 | 7.82** |

The largest departures from neutrality for each test, as determined by simulations or published critical values, are indicated in bold text. The labels African and European include all haplotypes, while the label European[-1] indicates all European haplotypes excluding a single ancestral haplotype/chromosome. n is the number of chromosomes, S is the number of segregating sites, θ_W_ is Watterson’s estimate of the population mutation rate based on S, and π is the nucleotide diversity.

1. (*)p-value < 0.05
2. (**)p-value < 0.01
3. (***)p-value < 0.00
