# Supplementary material for: Parallel Selection on TRPV6 in Human Populations
Source: PLoS One. 2008 Feb 27;3(2):e1686. doi: 10.1371/journal.pone.0001686 (PMC2246018; doi:10.1371/journal.pone.0001686)
Supplement: Table S3 — World Haplotype Frequencies for C157R, M378V and M681T (0.03 MB DOC) [file pone.0001686.s011.doc]

**Table S3. World Haplotype Frequencies for the C157R, M378V and M681T mutations**

Population Region TAT CGC TAC TGC CGT TGT

Stoneking Samples

Ethiopia Europe 85.48 14.52 0.00 0.00 0.00 0.00

German Europe 93.48 6.52 0.00 0.00 0.00 0.00

South_Afirca SubSahran Africa 36.36 63.64 0.00 0.00 0.00 0.00

Koragas India 100.00 0.00 0.00 0.00 0.00 0.00

Mullukurunan India 100.00 0.00 0.00 0.00 0.00 0.00

Mullukurumba India 100.00 0.00 0.00 0.00 0.00 0.00

CEPH Samples

Biaka_Pygmies SubSahran Africa 0.43 0.30 0.27 0.00 0.00 0.00

Mbuti_Pygmies SubSahran Africa 0.33 0.30 0.30 0.07 0.00 0.00

Mandenka SubSahran Africa 0.44 0.60 0.06 0.00 0.00 0.00

Yoruba SubSahran Africa 0.35 0.64 0.02 0.00 0.00 0.00

BanutN.E. SubSahran Africa 0.67 0.33 0.00 0.00 0.00 0.00

Bantu S SubSahran Africa 0.44 0.50 0.00 0.00 0.06 0.00

San SubSahran Africa 0.07 0.79 0.00 0.00 0.07 0.07

Mozabite North Africa 0.85 0.15 0.00 0.00 0.00 0.00

Bedouin Middle East 0.90 0.09 0.00 0.00 0.00 0.01

Druze Middle East 0.91 0.09 0.00 0.00 0.00 0.00

Palestin Middle East 0.94 0.06 0.00 0.00 0.00 0.00

Brahui Asia 0.96 0.04 0.00 0.00 0.00 0.00

Balochi Asia 0.96 0.04 0.00 0.00 0.00 0.00

Hazara Asia 0.98 0.02 0.00 0.00 0.00 0.00

Makrani Asia 0.98 0.02 0.00 0.00 0.00 0.00

Sindhi Asia 100.00 0.00 0.00 0.00 0.00 0.00

Pathan Asia 100.00 0.00 0.00 0.00 0.00 0.00

Kalash Asia 100.00 0.00 0.00 0.00 0.00 0.00

Burusho Asia 0.98 0.02 0.00 0.00 0.00 0.00

Han Asia 0.97 0.03 0.00 0.00 0.00 0.00

Tujia Asia 0.95 0.05 0.00 0.00 0.00 0.00

Yizu Asia 100.00 0.00 0.00 0.00 0.00 0.00

Miaozu Asia 0.95 0.05 0.00 0.00 0.00 0.00

Oroqen Asia 100.00 0.00 0.00 0.00 0.00 0.00

Daur Asia 100.00 0.00 0.00 0.00 0.00 0.00

Mongola Asia 100.00 0.00 0.00 0.00 0.00 0.00

Hezhen Asia 100.00 0.00 0.00 0.00 0.00 0.00

Xibo Asia 0.94 0.06 0.00 0.00 0.00 0.00

Uygur Asia 100.00 0.00 0.00 0.00 0.00 0.00

Dai Asia 100.00 0.00 0.00 0.00 0.00 0.00

Lahu Asia 100.00 0.00 0.00 0.00 0.00 0.00

She Asia 0.94 0.06 0.00 0.00 0.00 0.00

Naxi Asia 100.00 0.00 0.00 0.00 0.00 0.00

Tu Asia 100.00 0.00 0.00 0.00 0.00 0.00

Yakut Asia 100.00 0.00 0.00 0.00 0.00 0.00

Japanese Asia 0.98 0.02 0.00 0.00 0.00 0.00

Cambodia Asia 100.00 0.00 0.00 0.00 0.00 0.00

Papuan Oceania 100.00 0.00 0.00 0.00 0.00 0.00

NAN Melan Oceania 0.98 0.02 0.00 0.00 0.00 0.00

French Europe 0.93 0.07 0.00 0.00 0.00 0.00

French Basque Europe 0.83 0.17 0.00 0.00 0.00 0.00

Sardinian Europe 0.93 0.07 0.00 0.00 0.00 0.00

North Italian Europe 0.89 0.11 0.00 0.00 0.00 0.00

Tuscan Europe 0.94 0.06 0.00 0.00 0.00 0.00

Orcadian Europe 100.00 0.00 0.00 0.00 0.00 0.00

Adygei Europe 0.94 0.06 0.00 0.00 0.00 0.00

Russian Europe 0.98 0.02 0.00 0.00 0.00 0.00

Pima America 100.00 0.00 0.00 0.00 0.00 0.00

Maya America 0.98 0.02 0.00 0.00 0.00 0.00

Colombian America 100.00 0.00 0.00 0.00 0.00 0.00

Karitiana America 100.00 0.00 0.00 0.00 0.00 0.00

Surui America 100.00 0.00 0.00 0.00 0.00 0.00
