# Supplementary material for: Parallel Selection on TRPV6 in Human Populations
Source: PLoS One. 2008 Feb 27;3(2):e1686. doi: 10.1371/journal.pone.0001686 (PMC2246018; doi:10.1371/journal.pone.0001686)
Supplement: Table S4 — PCR primers for MALDI-TOF assay (0.03 MB DOC) [file pone.0001686.s012.doc]

**Table S4. PCR primers and MALDI-TOF SBE primers for non-synonymous sites C157R (rs4987657), M378V (rs4987667), and M681T (rs4987682).**

| **SNP** | **Forward PCR Primer** | **Reverse PCR Primer** | **Product size** |  |
| --- | --- | --- | --- | --- |
| rs4987657 | AGG TCA GAC TGC ACT GCA CAT | CTT GCC CAC CTT TCC AAC TG | 190bp |  |
| rs4987667 | GAG CAT TTT GAC TGT TCT TCT CC | ATG ATG ATA GCC CCA ATG ACA GT | 105bp |  |
| rs4987682 | AGG ATT TGG ACA AAG ACT CAG TG | CTC TGG GTG TTT GGT TTT TGT T | 291bp |  |
|  | **Extension Primer** | **Cleaved Primer Mass** | **Mass derived allele** | **Mass ancestral allele** |
| rs4987657 | TCA CCA AAG TAL ATG AGG TTG C | 3163.15 | 3460.37(A) | 3476.35(G) |
| rs4987667 | TCC CCC ATT AGL AAG CCT AC | 2458.65 | 2755.87(A) | 2771.85(G) |
| rs4987682 | AGG TAC TTC GAG ACL CTG AGG GC | 2530.69 | 2827.91(A) | 2843.89(G) |
