# Supplementary material for: Parallel Selection on TRPV6 in Human Populations
Source: PLoS One. 2008 Feb 27;3(2):e1686. doi: 10.1371/journal.pone.0001686 (PMC2246018; doi:10.1371/journal.pone.0001686)
Supplement: Table S5 — Table of PCR and sequencing primers (0.03 MB DOC) [file pone.0001686.s013.doc]

**Table S5. Table of PCR and sequencing primers.**

| **Sequence block** | **Forward Primer** | **Reverse Primer** | **Start** | **End** | **Annealing Tmp** | **Product size** |
| --- | --- | --- | --- | --- | --- | --- |
| *TRPV6*_2 | CAA TTG AAC TGT TTG CTC AGG AC | GTG CAT TTG TGT GTG GTG TTT A | 142295067 | 142295687 | 55 | 621 |
| *TRPV6*_3 | TGT AGG GCT GGT GAA AGA GG | GAA AGC AGG ACA CGG GTT TA | 142292462 | 142293090 | 60 | 629 |
| *TRPV6*_6 | GTC TTT GAG CCC ATG ACA TCT | ATT AAG CCC TAG AAG GAT TGC TC | 142284882 | 142285559 | 60 | 678 |
| *TRPV6*_7 | GAG GTG GTA CCC ATG TCC TTT | TGG TCC CTT CAT CTC AAT ATC AC | 142281675 | 142282470 | 55 | 796 |
| *TRPV6*_8 | CTT GCC AAC TCC ATC TTC AAT AA | GTA CCT TCA GTG ATG TGG CTC AG | 142278527 | 142279123 | 55 | 597 |
| *TRPV5*_1 | AGC TGA TGT TTG TAA AGC TGG AG | TGA ACA GTA ATC CGG GAA TCT AA | 142329663 | 142330251 | 55 | 589 |
| *TRPV5*_2 | AAT TTG GCA AAG ACA CTG AGC TA | GCA AAG GAA TGA AGT TTT CAG AG | 142327226 | 142327971 | 60 | 746 |

Start and end positions, on chromosome 7, are based on the March 2006 human reference sequence.
